# Signal Transduction by a Fungal NOD-Like Receptor Based on Propagation of a Prion Amyloid Fold

**DOI:** 10.1371/journal.pbio.1002059

**Published:** 2015-02-11

**Authors:** Asen Daskalov, Birgit Habenstein, Denis Martinez, Alfons J. M. Debets, Raimon Sabaté, Antoine Loquet, Sven J. Saupe

**Affiliations:** 1 Non-self recognition in Fungi, Institut de Biochimie et de Génétique Cellulaire, UMR 5095, CNRS—Université de Bordeaux, Bordeaux, France; 2 Institute of Chemistry & Biology of Membranes & Nanoobjects, CNRS, CBMN, UMR 5248, Pessac, France; 3 Laboratory of Genetics, Wageningen University, Droevendaalsesteeg, Wageningen, The Netherlands; 4 Institut de Nanociència i nanotecnologia, Departament Fisicoquímica, Universitat de Barcelona, Joan XXIII s/n, Barcelona, Spain; The Whitehead Institute for Biomedical Research, UNITED STATES

## Abstract

In the fungus *Podospora anserina*, the [Het-s] prion induces programmed cell death by activating the HET-S pore-forming protein. The HET-s β-solenoid prion fold serves as a template for converting the HET-S prion-forming domain into the same fold. This conversion, in turn, activates the HET-S pore-forming domain. The gene immediately adjacent to *het-S* encodes NWD2, a Nod-like receptor (NLR) with an N-terminal motif similar to the elementary repeat unit of the β-solenoid fold. NLRs are immune receptors controlling cell death and host defense processes in animals, plants and fungi. We have proposed that, analogously to [Het-s], NWD2 can activate the HET-S pore-forming protein by converting its prion-forming region into the β-solenoid fold. Here, we analyze the ability of NWD2 to induce formation of the β-solenoid prion fold. We show that artificial NWD2 variants induce formation of the [Het-s] prion, specifically in presence of their cognate ligands. The N-terminal motif is responsible for this prion induction, and mutations predicted to affect the β-solenoid fold abolish templating activity. In vitro, the N-terminal motif assembles into infectious prion amyloids that display a structure resembling the β-solenoid fold. In vivo, the assembled form of the NWD2 N-terminal region activates the HET-S pore-forming protein. This study documenting the role of the β-solenoid fold in fungal NLR function further highlights the general importance of amyloid and prion-like signaling in immunity-related cell fate pathways.

## Abstract 2

The fungus *Podospora anserina* uses a prion amyloid fold as a signal transduction device between a Nod-like receptor and a downstream cell death execution protein.

## Author Summary

Although amyloids are best known as protein aggregates that are responsible for fatal neurodegenerative diseases, amyloid structures can also fulfill functional roles in cells. In particular, the controlled formation of amyloid structures appears to be involved in different signaling processes in the context of programmed cell death and host defense. The [Het-s] prion of the filamentous fungus *Podospora anserina* is a model system in which the 3-D structure of the prion form has been solved. The [Het-s] prion works as an activation switch for a second protein termed HET-S. HET-S is a pore-forming protein that is activated when the [Het-s] prion causes its C-terminal domain to adopt an amyloid-like fold. The protein encoded by the gene adjacent to *het-S* is a Nod-like receptor (NLR) called NWD2. NLRs are immune receptors that control host defense and cell death processes in plants, animals, and fungi. We show that NWD2 can template the formation of the [Het-s] prion fold in a ligand-controlled manner. NWD2 has an N-terminal motif homologous to the HET-S/s prion-forming region; we find that this region is both necessary and sufficient for its prion-inducing activity, and our functional and structural approaches reveal that the N-terminal region of NWD2 adopts a fold closely related to that of the HET-S/s prion. This study illustrates how the controlled formation of a prion amyloid fold can be used in a signaling process whereby a Nod-like receptor protein activates a downstream cell death execution domain.

## Introduction

Proteins that assemble into amyloids are responsible for a variety of human neurodegenerative pathologies [1,2]. Yet, it has become clear that amyloids are not necessarily associated with disease [3]. So called functional amyloids have been involved in a variety of biological processes such as peptide hormone storage and release and cell envelope formation in microbes [4]. Amyloid polymers have a self-templating activity, allowing them to propagate as prions (infectious proteins) [5–7]. Prion proteins were first identified as causal agents of transmissible spongiform encephalopathies such as Creutzfeld-Jacob disease in humans [8]. Prions also exist in eukaryotic microbes, where they were initially described as non-Mendelian cytoplasmic genetic elements [9].

[Het-s] is a prion of the filamentous fungus *Podospora anserina* [10,11]. This prion is involved in a non-self recognition process common in filamentous fungi and termed heterokaryon incompatibility. Incompatibility corresponds to a cell death reaction that occurs when filaments of unlike strains undergo fusion. Heterokaryon incompatibility serves to limit fusion between genetically unlike individuals, in order to restrict transmission of deleterious plasmids and fungal viruses or to prevent conspecific parasitism [12,13]. The *het-s* locus exists as two incompatible alleles termed *het-s* (*small s*) and *het-S* (*large s*). A fusion between *het-s* and *het-S* strains results in a cell death reaction and leads to the formation, between the strains, of an abnormal contact line termed barrage [10]. *Het-s* strains exist as two epigenetic metastable states: the active [Het-s] prion state and the inactive [Het-s*] non-prion state. [Het-s] is infectious and is transmitted systematically during cell fusion. This prion is highly prevalent in nature; in a wild population, 90% of *het-s* isolates were found to carry [Het-s] [14]. HET-s and HET-S are highly homologous proteins of 289 amino acid in length, displaying a C-terminal prion forming domain (PFD), from residue 218 to 289 and a N-terminal pore-forming toxin domain termed HeLo [15,16]. The two proteins differ functionally in their respective HeLo domains. HET-s lacks pore-forming activity due to a specific amino acid substitution in that domain [15,17]. In turn, the HET-s and HET-S PFD regions are functionally equivalent and interchangeable.

The PFD adopts a specific amyloid β-solenoid fold with two rungs of β-strands per monomer delimiting a triangular hydrophobic core. Each rung is formed by a 21 amino acid pseudo-repeat and the two repeats are connected by a 15 amino acid flexible loop [18–20]. The β-solenoid structure is stabilized by two pairs of asparagine ladders and three salt bridges per monomer [18,20–22]. In the soluble state of HET-S/s, the PFD region is flexible and unstructured [15,16]. In the incompatibility reaction, the HET-s PFD converts the HET-S PFD region into the β-solenoid fold [17]. This conversion of the HET-S PFD region then leads to a refolding of the HET-S HeLo domain, which exposes a N-terminal hydrophobic -helix and acquires pore-forming activity [17]. In vivo, HET-S relocates from the cytoplasm to the cell membrane where it exerts toxicity [23].

**Fig 1:**
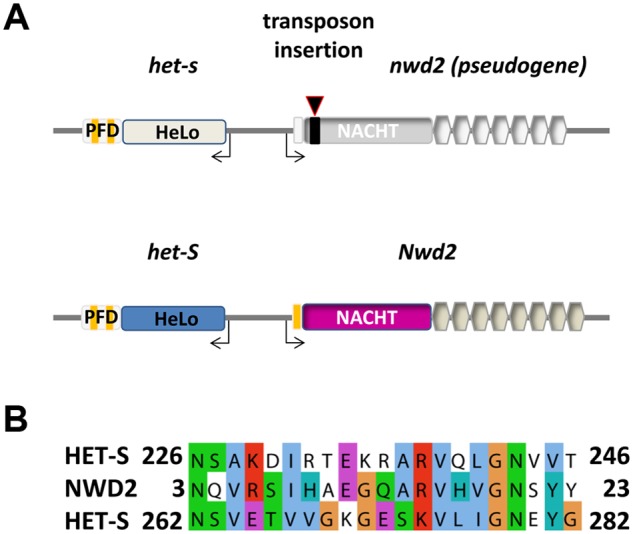
Genomic organization of the *Nwd2/het-S* locus and homology between the HET-S PFD region and the N-terminal region of NWD2. A. Gene structure at the *het-s/Nwd2* locus in wild-type *het-S* and *het-s* strains. NWD2 is encoded by the gene immediately adjacent to *het-S* in the genome. The two genes are divergently transcribed. Open reading frames are separated by 0.6 kb. In *het-s* wild-type strains, *nwd2* contains a *repa* transposon scar corresponding to an approximately 0.5 kb insertion, leading to premature stop codons; *nwd2* is thus in an inactive pseudogene form in all wild-type *het-s* strains (after [14,24]). B. Sequence alignment of the NWD2 N-terminal region (3–23) and the first (226–246) and second 21 amino acid repeat (262–282) of the prion-forming region of HET-S.

The gene immediately adjacent to *het-S* in the genome of *P*. *anserina* encodes a STAND (Signal Transduction ATPase with Numerous Domains) signal transduction protein termed NWD2 with a central NACHT domain and a C-terminal WD-repeat domain and displaying at its N-terminus a short region of homology with the elementary 21 amino acid repeats of the β-solenoid motif of the HET-S/s PFD (Fig. 1) [24]. STAND proteins are involved in gene regulation functions in bacteria and host defense and cell death in eukaryotes [25–29]. In particular, STAND proteins represent key components of the innate immunity system in metazoans and plants as Nod-like receptors and NBS-LRR proteins, respectively [30]. STAND proteins also have host defense and non-self recognition functions in fungi [31–34]. STAND proteins typically undergo ligand-induced oligomerization in response to ligand-binding to their C-terminal repeat domain, which in turn leads to activation of their N-terminal domain [30]. One of the best characterized STAND proteins is human APAF-1, the inducer of the intrinsic apoptosis pathway [35]. Binding of the cytochrome *c* to the C-terminal WD domain induces oligomerization of APAF-1 into a ring-shaped heptamer, in which the N-terminal CARD domains of the protein are clustered at the center of the ring. The spatial grouping of the CARD domains into a central hub allows binding and activation of caspase-9. We have proposed, based on the general oligomerization properties of STAND proteins, that upon recognition of a specific ligand in the WD repeat domain, NWD2 might undergo oligomerization via its NOD domain (nucleotide-binding and oligomerization domain), which would lead to the spatial clustering of N-terminal PFD-like regions [24]. This spatial grouping would then allow the cooperative folding of these regions into a HET-s—like β-solenoid fold and downstream activation of the HET-S HeLo toxicity domain. In this model, the HET-S toxin is the cell death execution module of the NWD2 receptor [36]. Of note is that *nwd2* is in an inactivated pseudo-gene form in all wild-type *het-s* isolates (Fig. 1) [14]. HET-S/[Het-s] incompatibility can occur in the absence of a functional *Nwd2* gene [16,37]. Thus, [Het-s] formation and [Het-s]/HET-S incompatibility occurs independently of NWD2. Cai and colleagues have recently shown that the HET-s PFD region and the N-terminal extension of NWD2 can functionally replace the PYD domain, operating in a prion-like signal transduction process between a human Nod-like receptor (NLRP3) and its cognate effector protein, thus providing direct support for the functional interaction of NWD2 and the HET-S/s PFD [38].

Herein, we have analyzed the NWD2/PFD interaction and use the ability to induce formation of the [Het-s] prion as reporter for the formation of a HET-S/s-like β-solenoid fold. Since the cognate ligand of NWD2 is currently unknown, we have set up an experimental system in which the WD-repeats of NWD2 have been replaced by closely homologous WD domains from proteins of the same multigene family and for which ligands are precisely known. We show that such chimeric NWD2 proteins specifically induce [Het-s] prion formation in vivo in response to their cognate ligands, thus providing evidence for heterologous prionization of [Het-s] by NWD2 variants. We then show that this activity of NWD2 is dependent on its N-terminal HET-S/s-like motif and that mutations that affect the β-solenoid fold reduce or abolish NWD2 prion-inducing activity. Next, we show that this templating activity of NWD2 can also occur in yeast, used as a remote heterologous host. We report that a NWD2(3–23) synthetic peptide forms amyloid fibrils with [Het-s] prion infectivity and present solid-state NMR data that indicates that NWD2(3–23) amyloids have a HET-S/s-like fold. We go on to show that a NWD2(1–30)-GFP (Green Fluorescent Protein) fusion protein is able to induce activation of HET-S toxicity. Our results show that NWD2 can act as a heterologous template for HET-S/s PFD transconformation and establish transmission of this prion fold as a mechanism of signal transduction by which a fungal Nod-like receptor activates a downstream cell death execution protein.

## Results

### Chimeric *nwd2* Alleles Induce [Het-s] Formation in a Ligand Dependent Manner

**Fig 2:**
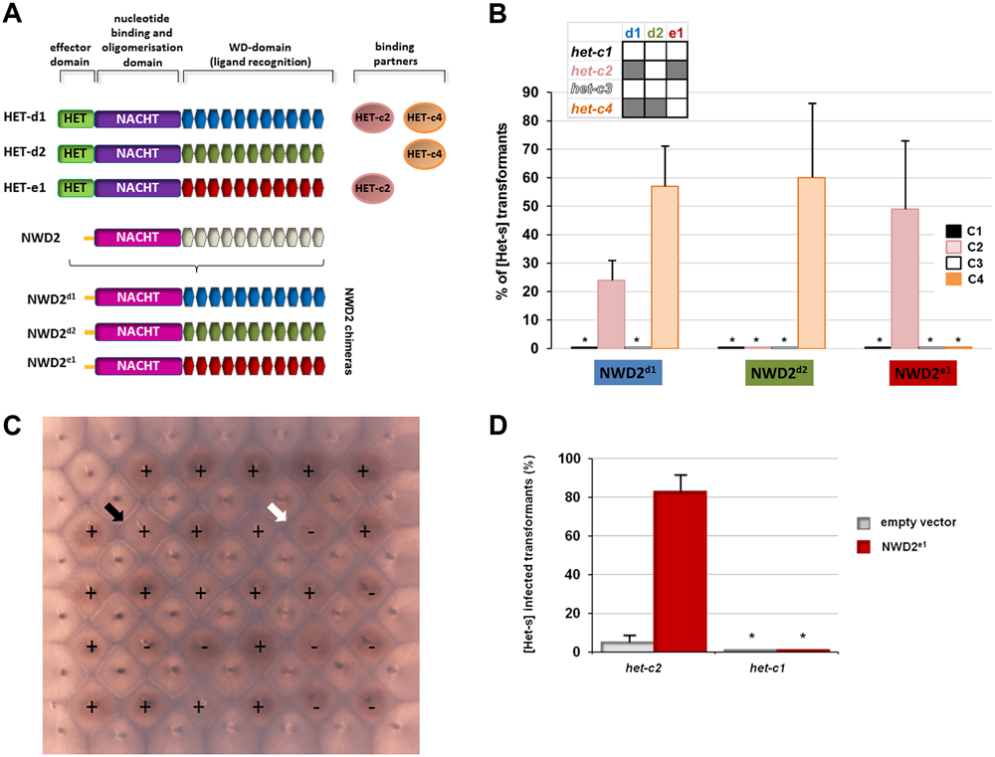
NWD2 chimeric alleles induce [Het-s] prion formation specifically in the presence of their cognate ligand. A. The domain organization of HET-d1, HET-d2, and HET-e1 are presented together with the HET-c variants they recognize and compared to the domain organization of NWD2. HET-d/e and NWD2 share a NACHT and a WD-repeat domain but differ in the N-terminal region, which corresponds to a HET, cell death—inducing domain in HET-d and HET-e (green box) and a region homologous to the PFD in NWD2 (yellow segment). In the bottom panel, the chimeric NWD2 variants that were constructed and in which the WD-repeat domain has been replaced by the corresponding domain from HET-d1, HET-d2, and HET-e1 are presented. B. Fraction of transformants converted to the [Het-s]-prion phenotype after transformation with different chimeric alleles of NWD2 (as noted). Results are given for four different *het-c* backgrounds (*het-c1*, *het-c2*, *het-c3*, and *het-c4*). In the inset, the interaction patterns between d1, d2 and e1 WD-domains and the different *het-c* alleles are recalled (after [47]). A grey box indicates that the *het-c* allele is recognized by the WD-domain; a white box indicates lack of interaction. Experiments have been carried out at least in triplicate and error bars are standard deviations; the asterisks denote that prion formation rate was zero. C. Raw data from a representative experiment is presented to illustrate how results were collected. Twenty-nine *ΔHsp104* [Het-s*] *het-c4* strains transformed with the *nwd2* ^d1^ chimeric allele (marked by + or-) were tested in barrage assays in confrontation to *het-S* testers (each transformant is surrounded by four *het-S* tester strains). Transformants presenting a barrage reaction to the *het-S* testers are score positive (+); transformants remaining [Het-s*] after transformation show no barrage reaction and are score negative (-). The black and white arrows point to examples of presence and absence of a barrage reaction, respectively. On this plate, 21 out of 29 tested strains acquired [Het-s] after transformation with the chimeric *nwd2* allele. D. NWD2^e1^ induces [Het-s] prion formation in a ligand-dependent manner in a wild-type strain for *PaHsp104*. Fraction of transformants converted to the [Het-s]-prion phenotype after transformation with *nwd2* ^e1^ in *het-c1* and *het-c2* backgrounds (wild-type for *PaHsp104*). Experiments have been carried out at least in triplicate and error bars are standard deviations; the asterisks denote that prion conversion rate was zero.

NWD2 is proposed to activate HET-S by templating of its PFD region into the β-solenoid fold [24,38]. We set out to test the ability of NWD2 to induce transconformation of the PFD. We have used [Het-s]-prion formation as a convenient and sensitive way to monitor the ability of NWD2 to template the PFD transconformation. The cognate ligands of the NWD2 WD-domain are unknown. Therefore, we decided to construct chimeric versions of NWD2 containing WD-repeat domains responding to known ligands. NWD2 is part of a large multigene family termed *nwd* comprising ten members which form a non-self recognition receptor repertoire in *P*. *anserina* [24,39–41]. Two genes in this family, *het-e* and *het-d*, have been previously characterized, and their cognate ligands are known [33,42–44]. HET-e and HET-d, also known as HNWD4 and HNWD5 respectively, recognize specific variants of a protein termed HET-C. HET-C is a glycolipid transfer protein which exists as many (at least 11) polymorphic variants in *P*. *anserina* [42,45–47]. Each *het-d* and *het-e* allele is characterized by a specific set of incompatible interactions with *het-c* alleles [33,43,47]. We created three chimeric alleles of NWD2 that we termed NWD2^d1^, NWD2^d2^, and NWD2^e1^, in which the WD-repeats of NWD2 were replaced by the homologous region from HET-d1, HET-d2, and HET-e1, respectively, each of which shows a specific interaction pattern with different allelic variants of HET-c (Fig. 2A and inset in Fig. 2B). The WD-repeat domain of the allelic variant HET-d1 (WD^d1^) recognizes both HET-c2 and HET-c4. WD^e1^ recognizes HET-c2 but not HET-c4, while conversely WD^d2^ recognizes HET-c4 but not HET-c2. None of the three WD-domains (WD^d1^, WD^d2^, and WD^e1^) interacts with HET-c1 or HET-c3. These WD-repeats (d1, d2, and e1) are highly homologous to the NWD2 WD-repeats (approximately 75% identity) [39].

These three chimeric alleles of *nwd2* were introduced by transformation into [Het-s*] *ΔPaHsp104* strains bearing different *het-c* alleles. Individual transformants were selected and then tested individually for the [Het-s] prion phenotype in barrage test against a *het-S* tester strain (Fig. 2B and 2C). Experiments were carried out in *ΔPaHsp104* strains because spontaneous [Het-s]-prion formation is very low in that background [48]. We found that each NWD2 chimera induces [Het-s] formation specifically in presence of the cognate ligand of the corresponding WD-domain. NWD2^d1^ induces [Het-s] in *het-c2* and *het-c4* backgrounds. NWD2^d2^ does so only in a *het-c4* background and NWD2^e1^ only in a *het-c2* background. All three chimeras were totally inactive in *het-c1* and *het-c3* backgrounds, as predicted. Activity of the NWD2^e1^ chimera was also tested in a wild-type background for *PaHsp104*. Again, the chimera presented [Het-s]-inducing activity specifically in the presence of HET-c2 but not HET-c1; prion-inducing activity was slightly higher than in the *ΔPaHsp104* background, but so was spontaneous prion formation (Fig. 2D). We conclude from these experiments that NWD2 chimeric alleles efficiently induce [Het-s]-prion formation specifically in the presence of the cognate ligand of their WD-repeat domain.

### Conservation of the NWD2 N-terminal Region

**Fig 3:**
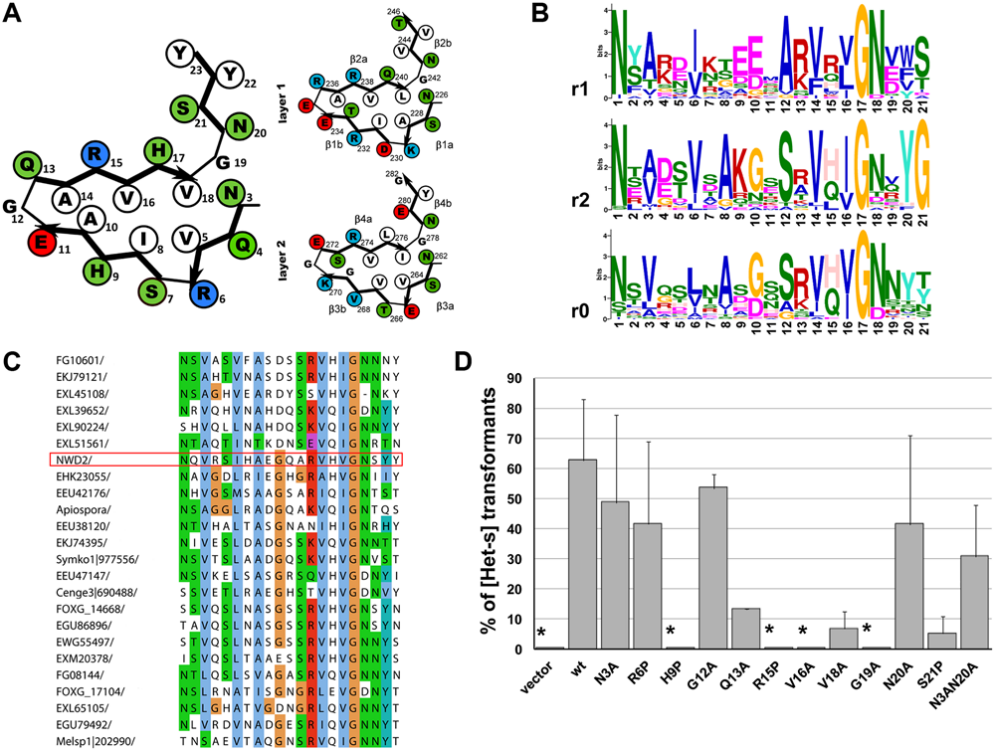
Mutation of conserved residues of the N-terminal region of NWD2 affect [Het-s]-inducing activity of chimeric *nwd2* alleles. A. An homology model of the NWD2(3–23) region based on the HET-s(218–289) β-solenoid fold (after [24]) is given together with a graphical representation of the structure of the two repeats of the HET-s PFD (after [18,19]). B. Comparison of the sequence conservation in repeats 1 and 2 of HET-s homologs (noted r1 and r2) and NWD2 homologs found in various pezizomycotina (noted r0). The consensus sequences were generated using MEME; the size of the letter reflects conservation; scale is given in informational content measured in bits. C. Alignment of the HET-s-like motif from NWD2 homologs found in various pezizomycotina. The NWD2 sequence is boxed in red. D. [Het-s]-inducing activity of wild-type and mutated *nwd2* ^e1^ alleles in a *ΔPaHsp104 het-c2* background. Experiments have been carried out at least in triplicate and error bars are standard deviations; the asterisks denote that prion conversion rate was zero.

NWD2 displays a region of homology to HET-S/s, and homology modelling suggests that the this region of NWD2 can adopt a HET-S/s-like fold (Fig. 3A) [24]. The HET-S/NWD2 genomic clustering is conserved in a wide range of fungal species: we could identify HET-S homologs in 55 sequenced fungal genomes, and in 46 cases a gene encoding a STAND protein with an N-terminal region homologous to the PFD region was found in an adjacent position (S1 Data). We compared the evolutionary conservation of the NWD2(3–23) motif (we term r0) with the r1 and r2 pseudo-repeats of HET-S homologs [21], (Fig. 3B and 3C), (S2 Data). The consensus sequence of the NWD2 motifs is closely related to the HET-S motif consensus and shows, in particular, conservation of the hydrophobic residues of the core (positions 3, 6, 8, 14, and 16), of the glycine residues in the β-arch positions (10 and 17), the ladder-forming asparagines (1 and 18), and the positive charge in position 13. There are, however, some differences between the NWD2 and HET-S motifs. In particular, there is a lower content in charged residues in the NWD2 motif sequences as compared to HET-S repeats (12.7% versus 23.6%, *p* < 5.10^–7^). Charged residues allow for complementary charges interactions during β-strand stacking of the r1/r2 pseudo-repeats but might be detrimental to homotypic NWD2 r0 stacking. The r0 NWD2-consensus motif is more closely related to the r2 than to the r1 motif (in particular at positions 8, 12, and 15). In NWD2, at the conserved salt-bridge position 4 and 9, charge complementary occurs with repeat 2 of HET-S, which might suggest that NWD2/PFD interaction occurs initially via an r0/r2 interface.

### Point Mutations Altering the β-solenoid Fold Affect Prion Induction Activity of NWD2

If NWD2 induces [Het-s] prion formation by adopting a β-solenoid—like fold, mutations affecting this fold are predicted to affect the [Het-s]-inducing activity. We have introduced in the N-terminal region of the NWD2^e1^ chimera point mutations analogous to mutations that were previously found to alter the β-solenoid fold, prion function of HET-s, and activation of HET-S [15,19,21,22]. These mutants were then tested for their [Het-s]-inducing activity in a *het-c2 ΔPaHsp104* background. We targeted the predicted β-strand regions, the hydrophobic core, the β-arch G residues and the N-ladders. We introduced a β-breaker proline mutation in each of the presumed β-strands of the predicted β-solenoid motif at R6, H9, R15, and S21 (Fig. 3A). Proline substitutions at position 9, 15, and 21 strongly affected NWD2^e1^ [Het-s]-inducing activity (Fig. 3D). R6P showed a limited effect, which might be related to the fact that R6 is located at the end of a predicted β-strand (Fig. 3A). Mutations of two Val residues of the predicted hydrophobic core (V16 and V18) also strongly affected [Het-s]-inducing activity. Mutations at the glycine residues G12 and G19 in the arch positions had differential effects. G12A had a modest effect on prion-inducing activity while G19A totally abolished function. Analogous substitutions in HET-s(218–289) affect [Het-s] activity differently, and only the mutations of the glycine residues analogous to G19 (G242A and G278A) have a strong effect [21]. In NWD2 homologs, G19 is more strongly conserved than G12 (Fig. 3B and 3C). Single mutants in the predicted Asparagine-ladders did not abolish activity, but a N3A N20A double mutant had a decreased activity. In HET-s(218–289), double mutations in the N residues at the equivalent positions (N226/N262 and N243/N279) are required to affect [Het-s] formation [21]. Together, these results indicate that the N-terminal HET-S/s-like motif of NWD2 is required for [Het-s]-inducing activity. Mutations predicted to affect the β-solenoid fold reduce or abolish this activity, consistent with the hypothesis that the (3–23) region of NWD2 adopts a PFD-like fold.

In different STAND proteins, ATP/ADP binding to the NACHT (or NB-ARC) NOD domain of is required for oligomerization [26]. A prediction of our model, based on the STAND protein paradigm, is that NWD2 activity should depend on the ability of the NACHT domain to bind ATP/ADP. We reasoned that inactivation of ATP/ADP binding should affect [Het-s]-inducing activity of NWD2. We generated four point mutants (K83N, K83R, D178A, and R214S) affecting respectively the P-loop motif (Walker A), the Walker B motif and the Sensor I region of the NACHT domain [49,50]. The position corresponding to the Sensor I was deduced from sequence alignment and a homology model to APAF-1. All four mutations totally abolished NWD2 [Het-s]-inducing activity supporting the notion that ATP/ADP binding to the NWD2 NACHT domain is required for [Het-s] inducing activity (S1 Fig.).

### NWD2 Induces [Het-s]_y_ Prion Formation in Yeast

**Fig 4:**
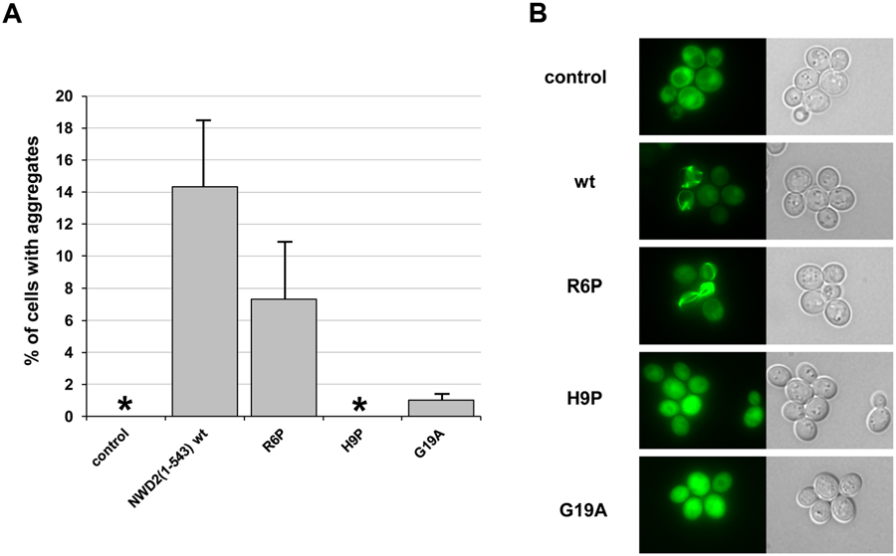
[Het-s]_y_-prion inducing activity of a NWD2(1–534) construct in yeast. A. A yeast strain expressing HET-s(218–289)-GFP was either transformed with an empty control vector or a plasmid, leading to expression of NWD2(1–534) and three different point mutants of NWD2(1–534) as noted. In each case, the fraction of cells containing dot or ring aggregates was determined. Experiments have been carried out at least in triplicates and error bars are standard deviations; the asterisks denote that aggregate formation rate was zero. At least 1,800 cells were counted for each construct, and the *p*-value of the difference to wild-type calculated as a two-tailed Fisher’s test was <0.001 in all cases. B. Representative fluorescence micrographs of the corresponding cells.

It was shown previously that prion propagation of the HET-s PFD can be achieved in a heterologous host, the yeast *Saccharomyces cerevisiae* [51]. This prion form of HET-s(218–289)-GFP was designated [Het-s]_y_. In order to strengthen the notion that NWD2 induces [Het-s]-formation by direct templating rather than through a process involving additional cellular partners, we determined whether [Het-s]-inducing activity could also be achieved in yeast (where no HET-s or STAND protein homologs are found). We expressed an N-terminal moiety of NWD2 encompassing the N-terminal motif and the NACHT domain and corresponding to the first exon of NWD2 under control of a constitutive promoter, together with HET-s(218–289)-GFP under control of a galactose inducible promoter. As show previously, under low galactose conditions (0.05% w/v), there was no spontaneous [Het-s]_y_ prion formation [51]. In the presence of NWD2(1–543), we observed formation of dot and ring aggregates of HET-s(218–289)-GFP, corresponding to the amyloid form of HET-s(218–289) (Fig. 4) [51]. Two β-breaker mutations (R6P and H9P) and mutation of the highly conserved G in the β-arch position (G19A), that affect NWD2^e1^ [Het-s]-inducing activity in *P*. *anserina*, also affected [Het-s]_y_-prion formation activity in yeast (Fig. 4). As previously observed in the *P*. *anserina* in vivo assay, the R6P mutation had less effect on [Het-s]_y_-inducing activity than H9P and G19A, but in this yeast model, the effect of the mutation was statistically significant.

We conclude that NWD2(1–534) induces [Het-s]_y_ prion formation in yeast and that this activity involves the N-terminal HET-S/s-like motif. This observation supports the notion that [Het-s]_y_ templating by NWD2 occurs directly without involving additional cellular partners, consistent with the results of Cai et al. showing that a functional PFD/NWD2(1–30) interaction can occur in mammalian cells [38].

### A NWD2(1–30)-GFP Fusion Protein Induces Prion Formation and Localizes to HET-s Aggregates

The N-terminal HET-S/s-like motif of NWD2 is required for [Het-s]-inducing activity. We next tested whether this region is sufficient for prion induction. We have expressed in *P*. *anserina* a NWD2(1–30)-GFP fusion protein encompassing the 3–23 motif in a *Δhet-s* background and in a [Het-s*] *ΔHsp104* background, from a strong constitutive promotor. The NWD2(1–30)-GFP was able to efficiently induce [Het-s] formation in [Het-s*] *ΔHsp104* strains and to propagate as a prion in *Δhet-s* strains (Table 1). The same assays were repeated with eight point mutants at various positions of the r0 motif tested previously in the context of the NWD2 chimera. Several point mutations abolished [Het-s]-inducing and propagation ability (H9P, R15P, V16P, V18P, and G19A) as previously observed for the NWD2 chimeras. G12A and R6P had more modest effects, also consistent with previous results. The effect of N20A was only observed in the prion propagation assay but not in the prion induction assay. The effect of the different mutations in the NWD2(1–30)-GFP context are largely correlated with the results obtained with the chimeric full-length *nwd2* alleles. We conclude from this experiment that NWD2(1–30)-GFP is sufficient to induce [Het-s] formation in vivo and that this prion-inducing activity is sensitive to point mutations predicted to affect the β-solenoid fold. The difference in [Het-s]-inducing activity was not due to lack of expression of the point mutants, as all analyzed mutants were expressed as detected by western-blotting (S2 Fig.).

**Table 1:** Prion-inducing and prion propagation ability of wild-type and mutant NWD2(1–30)-GFP.

	[Het-s] Prion Induction in Δ*Hsp104* [Het-s*]			[Het-s] Propagation in *Δhet-s*		
	[Het-s] infected /total tested	% of [Het-s] infected	*p*-value*	[Het-s] infected/total tested	% of [Het-s] infected	*p*-value*
NWD2(1–30)	69/90	80%	-	16/19	84%	-
R6P	30/60	50%	<0.02	1/14	7%	<0.001
H9P	0/60	0	<0.001	0/27	0	<0.001
G12A	14/60	23%	<0.001	1/11	9%	<0.001
R15P	0/60	0	<0.001	0/14	0	<0.001
V16A	0/60	0	<0.001	0/15	0	<0.001
V18A	0/59	0	<0.001	1/10	10%	<0.001
G19A	0/60	0	<0.001	0/16	0	<0.001
N20A	49/59	83%	n.s.	1/15	6%	<0.001

*two-tailed Fisher’s test for significance of the difference to wild-type

**Fig 5:**
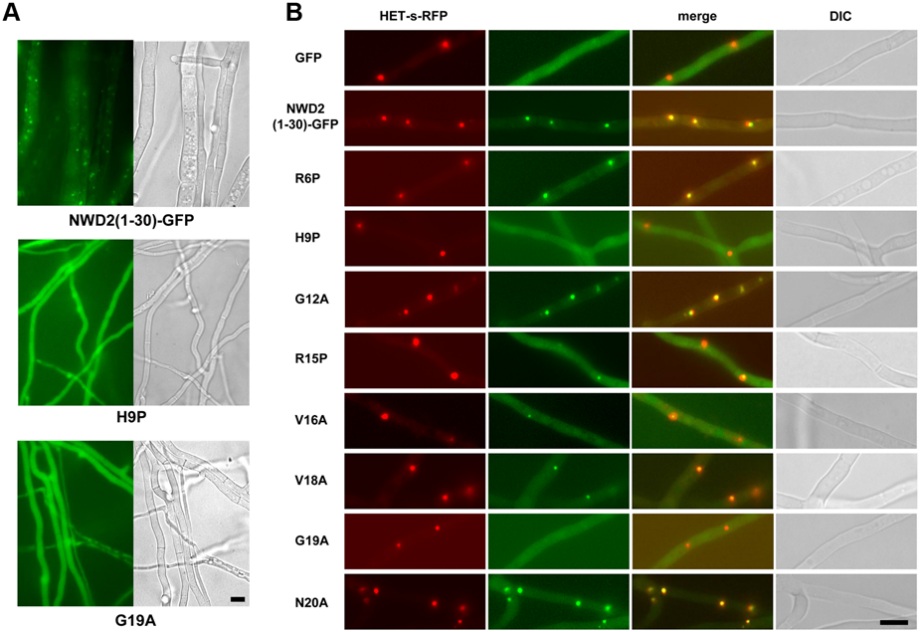
NWD2(1–30)-GFP localizes to HET-s-RFP prion aggregates. A. NWD2(1–30)-GFP, NWD2(1–30)-GFP H9P and NWD2(1–30)-GFP G19A were expressed *Δhet-s* strain. Wild-type but not mutant fusion proteins formed dot-like aggregates, scale bar is 5 μ. B. Co-expression of HET-s-RFP with GFP, NWD2(1–30)-GFP and eight point mutants of NWD2(1–30)-GFP, scale bar is 5 μ.

Next, we analyzed distribution of NWD2(1–30)-GFP in *Δhet-s* strains. NWD2(1–30)-GFP formed dot-like foci but the H9P and G19A point mutants did not (Fig. 5A). We have then analyzed co-expression of NWD2(1–30)-GFP with HET-s-RFP (Red Fluorescent Protein) (Fig. 5B). We found that NWD2(1–30)-GFP dots co-localized with HET-s-RFP aggregates. We also tested the ability of the eight point mutants to form dots and localize to HET-s aggregates. H9P and G19P mutants were unable to form dots and showed no co-localization with HET-s while for other mutants a partial or total ability to incorporate HET-s aggregates was retained. R6P and G12A which are only modestly affected for prion inducing activity still show co-localization. In certain mutants affected for prion-inducing activity like R15P co-localization was decreased but still detectable. We conclude from this experiment that NWD2(1–30)-GFP co-localizes with HET-s-RFP and that mutation predicted to affect the β-solenoid fold and abolishing prion inducing activity affect co-localization with HET-s aggregates.

### NWD2(1–30)-GFP Leads to Incompatibility with HET-S

So far in this study, the NWD2 templating activity was analyzed by monitoring [Het-s]-formation rather than HET-S activation. Since the HET-s and HET-S PFD regions are functionally identical and interchangeable, [Het-s] formation can be considered as a reliable proxy for HET-S activation. Previous studies however suggests that HET-S activation function is more sensitive to mutation than [Het-s]-inducing activity [19,21]. We chose to determine whether NWD2(1–30)-GFP aggregates are indeed able to activate HET-S. To that end, transformants displaying NWD2(1–30)-GFP aggregates were confronted with a [Het-S] strain, there was formation of a barrage reaction between the strains (S3 Fig.). This barrage reaction was not observed against a [Het-s*], [Het-s], or *Δhet-s* strain, indicating that NWD2(1–30) leads to heterokaryon incompatibility with HET-S (not shown). The H9P and G19A mutations abolished this incompatibility to [Het-S] (S3 Fig.).

**Fig 6:**
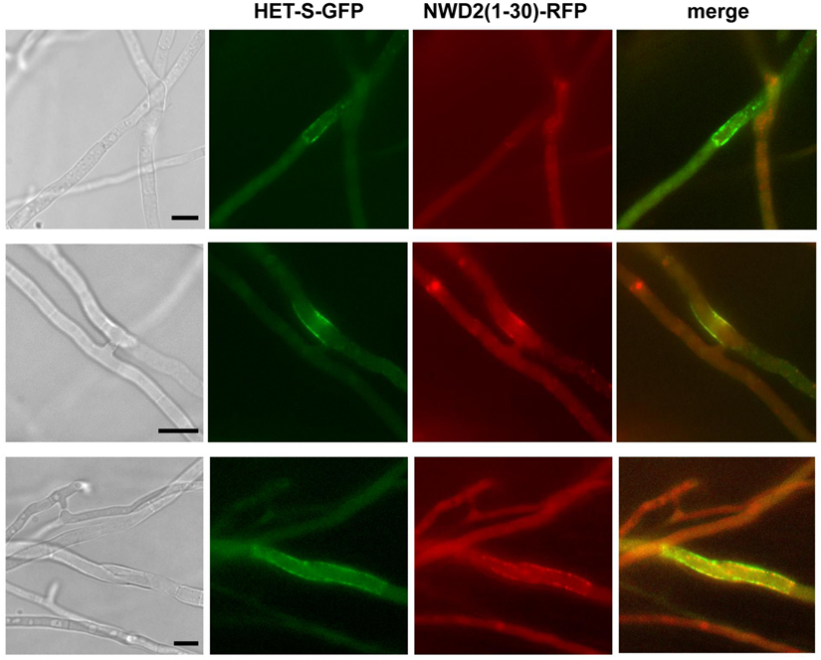
NWD2(1–30)-RFP causes re-localization of HET-S-GFP to the cell periphery. A strain expressing NWD2(1–30)-RFP was confronted to a strain expressing HET-S-GFP and heterokaryotic fusion cells were analyzed by fluorescence microscopy. Panels correspond from left to right to the bright field, GFP, RFP, and merged images. Note that HET-S-GFP localizes to the cell periphery and shows partial co-localization with NWD2(3–23)-RFP. Scale bar is 5 μ. The presented zoomed images are given in the full field from which they were extracted in S4 Fig.

We have previously reported that the [Het-s]/HET-S incompatibility reaction leads to re-localization of HET-S from the cytoplasm to the plasma membrane region upon activation of the pore-forming state of the HeLo domain [17,23]. We have therefore analyzed the localization of HET-S in the context of NWD2(1–30)/HET-S incompatibility. A strain expressing NWD2(1–30)-RFP was confronted to a HET-S-GFP strain, an localization of HET-S in fusion cells undergoing cell death was analyzed as done previously for [Het-s]/HET-S incompatibility [23]. We found that, as described previously in the [Het-s]/HET-S incompatibility context, HET-S was located in the plasma membrane region in the presence of NWD2(1–30)-RFP (Fig. 6, S4 Fig.). In these assays, NWD2(1–30) is functionally equivalent to [Het-s], this construct is able to induce [Het-s] prion formation but also to trigger incompatibility to HET-S associated with re-localization of HET-S to the cell membrane.

### A NWD(3–23) Peptide Forms Prion Amyloids In Vitro

**Fig 7:**
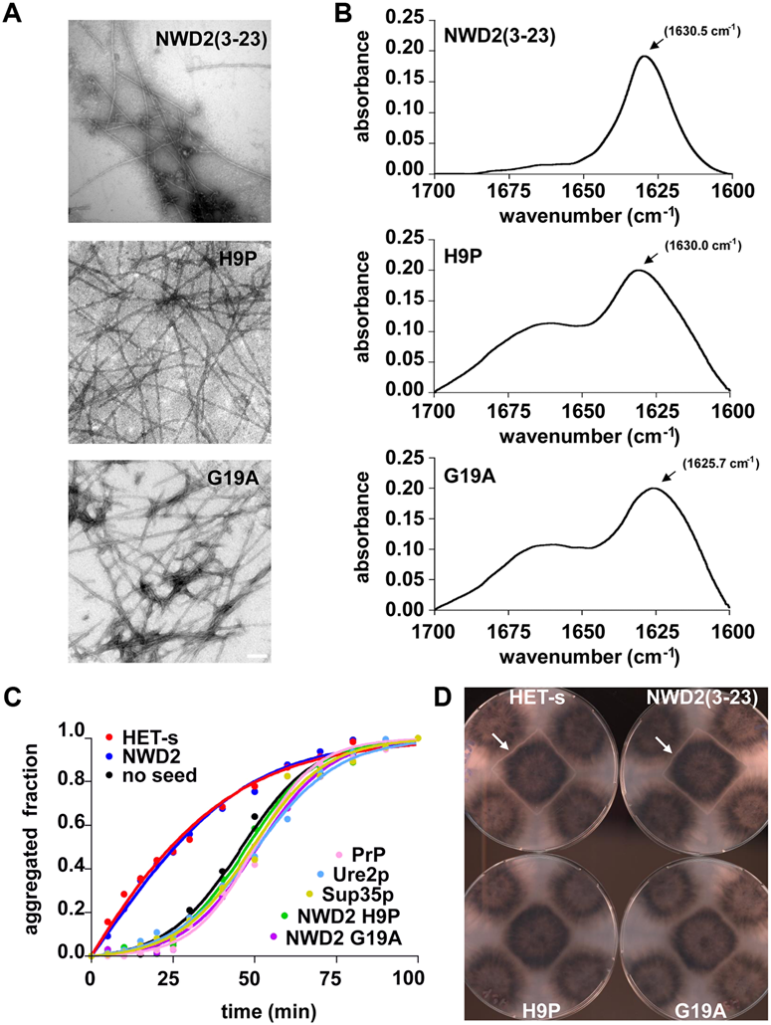
A 21 amino acid synthetic NWD2(3–23) peptide forms amyloid fibrils with prion infectivity. A. Electron micrograph of negatively stained NWD2(3–23) fibrils and of the H9P and G19A variants, scale bar is 100 nm. B. FTIR spectrum of NWD2(3–23) fibrils and of H9P and G19A variants. C. Kinetics of aggregation of 15 μM HET-s(218–289) at pH 7 and 37°C under high agitation (1400 r.p.m.). The aggregated fraction of protein is represented as a function of time. The aggregation reactions have been performed in absence of seeds and in presence of 1.5 μM of pre-aggregated amyloids as marked. D. Representative examples of the outcome of transfection of [Het-s*] with the different fibril types. Four transfected strains are tested on each plate and the tester strain in the center of the plate is [Het-S]. Note that only NWD2(3–23) and HET-s(218–289) fibrils convert strains to the [Het-s] phenotype (detected by formation of a barrage reaction to the central [Het-S] tester), full results are reported Table 2.

Next, we determined whether the N-terminal r0 motif of NWD2 is capable of amyloid formation in vitro and if so if such fibrils possess [Het-s]-templating activity. To that end, we generated a synthetic peptide encompassing just the 21 amino acid NWD2(3–23) sequence. The NWD2(3–23) peptide assembled spontaneously into fibrils (Fig. 7A). These fibrils appeared composed of single protofilaments of about 5 nm, as previously observed for HET-s(218–289) fibrils [52,53]. Next, we have analyzed the fibril structure by FTIR (Fourier Transform InfraRed spectroscopy). In FTIR, these fibrils show a strong amide I band at 1630 cm^-1^ generally attributed to a parallel β-sheet structure (Fig. 7B) [54,55]. Then we found that NWD2(3–23) fibrils are able to specifically seed in vitro fibrillization of HET-s(218–289), (Fig. 7C). To assess prion infectivity of these fibrils, NWD2(3–23) amyloids were then transfected into [Het-s*] strains, the fibrils showed high levels of prion infectivity (Fig. 7D, Table 2). This 21 amino acid peptide is the shortest fragment reported to display [Het-s]-infectivity. Mutant forms of the peptide, NWD2(3–23) H9P and G19A were still capable of fibril formation in vitro but these fibrils showed a different FTIR spectrum, were unable to seed HET-s(218–289) aggregation in vitro and had a strong reduction in prion infectivity (Fig. 7, Table 2). We have also constructed a chimeric version of HET-s(218–289) in which we replaced the two 21 amino acids pseudo-repeats of HET-s by the corresponding NWD2(3–23) motif. Such HET-s^NWD2(3–23)^ fibrils were also able to induce [Het-s]-formation in vivo (Table 2). These results indicate that the 21 amino acid motif NWD2(3–23) can functionally substitute for the pseudo-repeats in the HET-s(218–289) context for prion amyloid formation.

**Table 2:** Transfection of [Hets*] strains with wild-type and mutant NWD2(3–23) peptides.

Peptide	[Het-s] Infected/Total Tested	% of Prion Formation
none	1/44	2.3%
NWD2(3–23)	68/68	100%
NWD2(3–23) H9P	3/68	4.4%
NWD2(3–23) G19A	4/68	5.8%
HET-s(218–289)	68/108	62.9%
HET-s(218–289)^NWD2^	15/36	41.7%

We conclude from these experiments that NWD2(3–23) is capable of forming fibrils with [Het-s]-prion infectivity in vitro. Since [Het-s]-prion infectivity has been found to be correlated with the ability to adopt the HET-s β-solenoid fold [19,21,22,52,56–58], this result suggests that the NWD2(3–23) peptide adopts a HET-s-like fold.

### Solid-State NMR Suggest That NWD2(3–23) Adopts a HET-S/s-Like β-solenoid Fold

**Fig 8:**
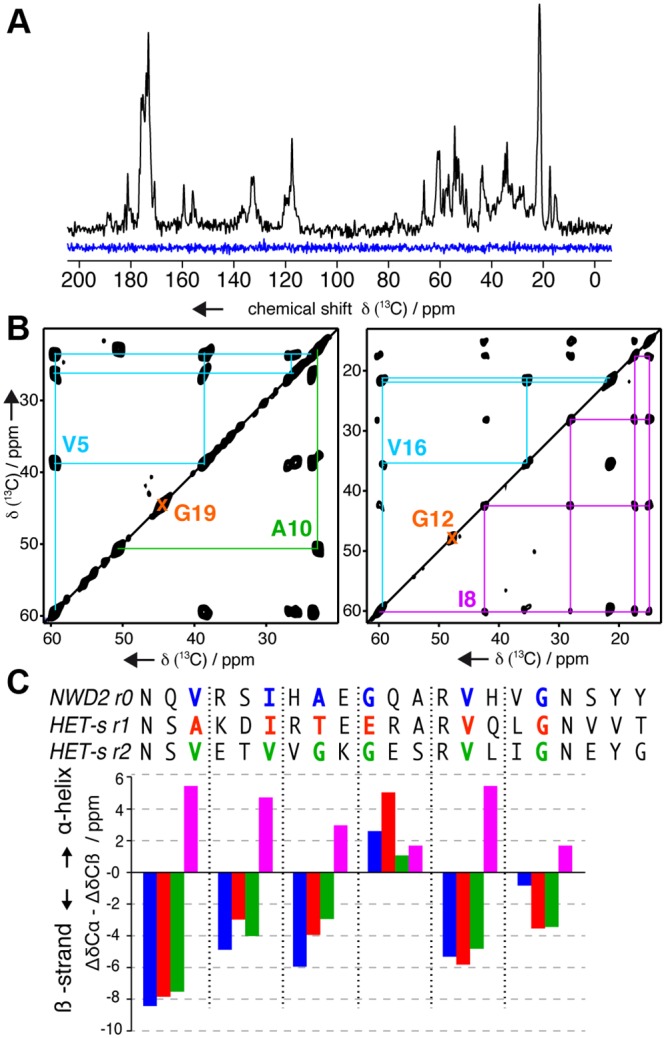
Conformational analysis of NWD2 fibrils by solid-state NMR. A. Solid-state NMR 13C spectra on natural abundance NWD2(3–23), recorded using a cross-polarization (black) and INEPT (blue) polarization transfers. B. Two-dimensional solid-state NMR 13C-13C correlation spectra of NWD2(3–23) fibrils, isotopically 13C labeled on Val5, Ala10, Gly19 (left) and Ile8, Gly12, Val16 (right). 13C resonance assignment of each amino acid is illustrated by lines: blue for Val, orange for Gly, green for Ala and pink for Ile. C. Comparison of the secondary structure encoded in the secondary chemical shift of NWD2 r0 (13C labeled residues in blue) with HET-s r1 (red) and r2 (green). Negative or positive values indicate β-strand or α-helix conformation respectively. Illustrated in pink are the hypothetical secondary chemical shifts for each residue of NWD2 in α-helical conformation [61,62].

To reveal the NWD2(3–23) amyloid conformation, we recorded one- and two-dimensional solid-state NMR spectra on NWD2(3–23) fibrils. Cross-polarization (CP) based solid-state NMR experiments allow for a polarization transfer between atoms in a rigid conformation, whereas Insensitive Nuclei Enhanced by Polarization Transfer (INEPT)-based transfer steps involve mobile segments. Comparing a one-dimensional CP- versus an INEPT-based ^13^C detected spectrum recorded on natural abundance NWD2(3–23) fibrils, we found NWD2(3–23) to adopt a rigid conformation throughout the entire peptide sequence (Fig. 8A). In contrast to the well-resolved CP-based spectrum, no signal is present in the INEPT-based spectrum, indicating the absence of any mobile residues. The narrow ^13^C line width ranging between 0.5–0.7 ppm at 7kHz magic-angle spinning (MAS) (63–88 Hz on a 500 MHz proton frequency spectrometer) shows that the protein has arranged in a highly ordered 3-D structure, comparable to HET-s(218–289) amyloids [59].

We further performed two-dimensional solid-state NMR to access the secondary structure of NWD2(3–23) in amyloid fibrils. We recorded ^13^C-^13^C correlation spectra on two NWD2(3–23) fibrils, ^13^C labeled on three residues: Val5/Ala10/Gly19 and Ile8/Gly12/Val16 respectively (Fig. 8B). Solid-state NMR secondary chemical shifts [60,61] are indicative for the secondary structure elements. We therefore compared the secondary chemical shifts of the corresponding residues in HET-s repeat 1 and 2 to those of NWD2(3–23). For all six tested residues (Val5, Ile8, Ala10, Gly12, Val16, and Gly19), situated at key positions in the β-solenoid structure [18], we found a highly similar secondary structure revealed by comparable secondary chemical shifts (Fig. 8C). Taken together, our solid-state NMR data consistently indicate that NWD2(3–23) adopts a rigid HET-S/s-like fold.

### The WD-Repeats Region of *nwd2* Is Highly Polymorphic in Natural Populations

We have constructed artificial alleles of *nwd2* that respond specifically to HET-c variants but the nature of the cognate ligand(s) of the WD-domain of wild-type *nwd2* is unknown. As an initial step to approach the question of the nature of the NWD2 ligand(s), we have analyzed the variability of the ligand binding WD-domain of *nwd2* in a natural population.

**Fig 9:**
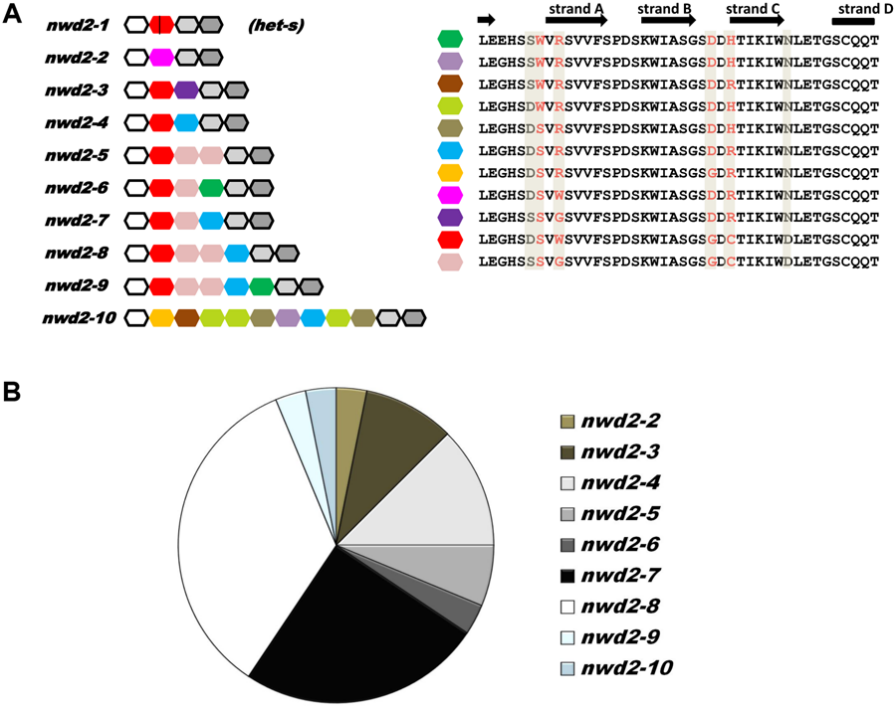
The NWD2 WD-domain is highly polymorphic in wild populations. A. Graphical representation of the WD-repeat domain of different *nwd2* alleles identified in wild population from Wageningen. Each 42 amino acid WD-repeat is represented by a hexagonal box. The three fixed repeats common to all alleles are given in white, light and dark grey while the variable repeats are colored. The vertical bar in the second repeat of the *nwd2–1* allele found in all *het-s* strains represents a stop codon. On the right, amino acid sequence of the different repeats are given, the four highly variable positions under positive diversifying selection are given in red and boxed in grey. Other polymorphic positions are boxed in grey. The secondary structure of each repeat is recall; variable loops at the WD-domain β-propeller binding surface are located between strand D and A, and B and C respectively. B. Pie chart of the *nwd2* allele distribution in the 32 analyzed wild-type *het-S* strains.

The WD-region coding region was analyzed in a collection of 79 wild-type *P*. *anserina* strains corresponding to a previously characterized population collected around Wageningen, The Netherlands [62]. The WD-domain is composed of two types of repeats, (1) fixed repeats located at the border of the repeat region and (2) variable repeats located centrally in the repeat array and that are exchanged in intragenic and extragenic recombination events and undergo concerted evolution in the different members of the *nwd* gene family [39,41]. As the result of concerted evolution, these repeats are highly similar within a repeat array and between repeat arrays in the different *nwd*-genes, we term them canonical repeats. The repeats are essentially identical except for four codon positions that are subjected to positive diversifying selection. These positions are predicted to be located at the interaction surface of the β-propeller structure of WD-domain. In 47 analyzed *het-s* strains, a single allele with four WD-repeats was found (three fixed repeats and one canonical repeat). In all sequenced strains, the second repeat was interrupted by a stop codon. In sharp contrast, in *het-S* strains there was extensive polymorphism. In the 32 analyzed *het-S* strains, nine different *nwd2* alleles were identified (Fig. 9A). Polymorphism corresponded to repeat number variation and polymorphism at the hypervariable sites of the different repeats. We could identify 11 different repeat types that were assorted differently in alleles ranging from four to 12 repeats (Fig. 9A, S1 Table). In Fig. 9B the frequency of the different alleles in the population is given. Two alleles *nwd2–7* and *nwd2–8*, displaying respectively six and seven repeats, were highly abundant in the population and together account for more than half of the analyzed population. Apparently, the repeat array evolved by repeat duplication and re-shuffling events, a situation previously observed for *het-e* and *het-r* alleles in an experimental evolution setting [41]. In other members of the gene family, this extensive polymorphism in the repeat domain is associated with difference in ligand binding patterns, making it likely that different *nwd2* alleles have different ligand binding properties.

We chose to further analyze the most frequent allele, *nwd2–8*, which accounts for one-third of the analyzed population. Since the WD-repeats array of all members of the *nwd* family are highly homologous and because two genes of this family bind HET-c, we ask whether HET-c could be also be ligand for *nwd2*. The *nwd2–8* allele were tested for the ability to induce [Het-s] in three different *het-c* backgrounds [47]. As previously described for the chimeric alleles, *nwd2–8* was transformed in *ΔHsp104* [Het-s*] containing different *het-c* alleles and transformants were tested in barrage assays for presence of the [Het-s] prion phenotype. No prion-inducing activity was detected for *nwd2–8* in any of the tested *het-c* backgrounds (S2 Table). These experiments suggest that neither of these HET-c variants is a ligand for NWD2–8. If HET-c1 or c2 were able to activate NWD2–8 and trigger HET-S toxicity, then these allele combinations would be expected to be absent in natural isolates because unviable. In fact the opposite is true: wild-type *nwd2–8* strains display *het-c1 or het-c2-*type alleles at the *het-c* locus (S1 Table). This observation is thus consistent with the experimental results that suggest that neither *het-c1* nor *het-c2*-type alleles are ligands for the frequent *nwd2–8* allele.

## Discussion

[Het-s] triggers programmed cell death by activating pore-forming activity of HET-S [17]. This activation relies on the templating of the β-solenoid fold in the C-terminal prion forming region of HET-S. This β-solenoid templating in turn leads to refolding on the N-terminal HeLo pore-forming domain. We have proposed, based on sequence analyses and homology modeling, that NWD2, a Nod-like receptor encoded by the neighboring gene, possesses [Het-s]/HET-S-templating activity [24]. In a study on the general role of prion-like signaling by immune complexes, Cai and colleagues have recently shown that the HET-s PFD and the N-terminal HET-S/s-like motif of NWD2 allow for signal transduction when attached to a human NLR and its effector protein, respectively NLRP3 and ASC [38]. We here present functional evidence of the prion templating activity of NWD2 in vivo in *Podospora anserina*. We show that versions of NWD2 engineered to respond to known ligands efficiently induces [Het-s]-formation in a ligand-dependent manner. We also show that the N-terminal region of NWD2 is both necessary and sufficient for prion-inducing activity. While full-length versions require a ligand for inducing [Het-s] formation, shorter versions lacking the WD-repeat region bypass the need for ligand binding, at least when strongly expressed in yeast. This is consistent with the inhibitory role of the WD-domain on oligomerization described in other STAND proteins like APAF-1 [26]. The minimal NWD2(1–30)-GFP fusion protein assembles spontaneously and acquires [Het-s]-inducing activity without a NACHT oligomerization domain. In this setting, amyloid assembly occurs spontaneously and not as a ligand-regulated process. In the study by Cai et al., in mammalian cells, NWD2(1–30) was necessary but not sufficient for conversion of the HET-s PFD [38]. Possibly the expression level was insufficient to allow for spontaneous prionization of NWD2(1–30), or else the mammalian cell context did not allow for this spontaneous aggregation.

Our results and the work by Cai et al. strongly support the model for NWD2/HET-S as a two-component system in which the signal transduction process from NLR to effector cell death execution domain involves propagation of an amyloid prion fold [38]. These results are best explained by proposing that ligand-induced oligomerization of NWD2 allows for spatial clustering of the HET-S/s-like motif and provides conditions for cooperative nucleation of the β-solenoid fold that can then template HET-S PFD transconformation and toxicity.

### NWD2(3–23) as a Minimal Prion Motif

NWD2 is able to template [Het-s] prion formation. Heterologous prion templating can occur between orthologous prion proteins in the case of species barrier crossing in mammalian and fungal prions [63,64]. The situation we describe here differs by the fact that seeding occurs between non-orthologous proteins that do share a homologous motif. Heterologous prion seeding also represents the physical basis of [*PIN* ^+^]-induced [*PSI* ^+^] prion formation in yeast [65]. In this case, templating occurs between proteins that share an amino acid composition bias but no detectable homology [64].

The assembled form of the NWD2(3–23) peptide awaits full structural characterization but several lines of evidence support the hypothesis that this region adopts a HET-s like fold. First, the region is homologous to the HET-s elementary repeats and predicted by homology modeling to be able to adopt the β-solenoid fold [24]. Also, NWD2 is able to induce [Het-s] prion formation, and [Het-s] templating activity has been correlated with formation of the β-solenoid fold [19,21,52,56–58]. Mutations that are predicted to affect the β-solenoid fold affect [Het-s] prion-inducing activity of NWD2 in vivo and in vitro. Finally and importantly, solid state NMR characterization of NWD2(3–23) fibrils is fully consistent with a HET-S/s-like fold. The characteristic β-strand distribution and the essential glycine arch of the HET-s(218–289) β-solenoid fold [18,22] is reflected in the fold of NWD2(3–23). Five residues situated in the three solenoid core β-strands and the critical glycine at the arch position reveal a very similar secondary structure fingerprint. In addition, all residues of NWD2(3–23) peptide are in a highly ordered and rigid conformation, consistent with the proposition that the peptide adopts a HET-S/s-like fold.

The HET-s(218–289) PFD is 72 amino acid in length [16]. The β-solenoid amyloid fold is characterized by the stacking along the fibril axis of two 21 amino acid pseudo-repeats connected by a flexible loop of 15 amino acids [18]. The second repeat is followed by a conserved and functionally important aromatic loop [21]. Each of these structural elements was found to be required for proper formation of the β-solenoid fold and/or prion activity [19,21,22]. The homology between the NWD2 N-terminal region and the HET-S/s PFD region is limited to the β-strand region, and importantly, NWD2 contains only a single repeat of the 21 amino acid motif [24]. Yet, it appears that this region is able to adopt a β-solenoid—like fold in vivo (in the context of full-length NWD2) and in vitro as isolated synthetic peptide. To our knowledge, the NWD2(3–23) peptide is the smallest molecular entity found to display prion inducing activity. This indicates that although the HET-s(218–289) prion structure is relatively complex [18,22], a single copy of the 21 amino acid repeat is sufficient for prion templating, demonstrating that the prion templating relies strictly on the β-strand regions (and does not necessitate either the flexible loop, the C-terminal aromatic loop or a repetition of the 21 amino acid motif). In addition, it appears that all the structural information for acquisition of a HET-s-like fold is contained in this 21 amino acid sequence. We have identified two point mutation that abolish [Het-s]-inducing activity of NWD2. One, H9P, is a β-breaker mutation in the second predicted strand; the other, G19A, is located in the critical glycine residue in the β-arch position of the motif. Interestingly, these mutations do not abolish amyloid polymerization of the NWD2(3–23) peptide but rather lead to formation of amyloids with an alternate structure (based on FTIR), with altered prion inducing and in vitro templating activity. An equivalent mutation of a glycine of an arch-position of HET-s(218–289) also leads to formation on an alternate non-infectious amyloid structure [21].

### On the Nature of the *nwd2* Ligand

Mammalian Nod-like receptors and plant NBS-LRR mediate host defense in response to non-self [66]. The defense response often takes the form of a “scorched earth” policy, a localized programmed cell death reaction that prevents further pathogen invasion [67,68]. NWD2 is part of a large gene family that control various forms of programmed cell death in fungi [69], and we have previously proposed that these fungal NLR homologs may function as immune receptors [31,34]. One may venture to speculate on the nature of the ligand that controls activation of NWD2 and induction of HET-S-mediated cell death. The NWD2 ligand could, in principle, correspond to modified self (as is generally the case in effector-triggered immunity in plants) or to a non-self cue (as in MAMP-triggered immunity in mammals), [66]. The HET-d and HET-e genes, which belong to the same gene family, recognized modified self (in the form of variants of HET-c), [47]. It is proposed that HET-c might be a pathogen-effector target placed under surveillance of the HET-d and HET-e NLRs [47]. For NWD2, genetic and experimental evidence suggest, however, that the binding partner is not HET-c (at least in the case of the frequent *nwd2–8* allele). This possibility of a NWD2/HET-c interaction cannot, however, be formally ruled out at present. This would require the combinatorial testing of the interaction of the nine identified *nwd2* alleles with the 11 known *het-c* alleles. The WD-region of NWD2 is subject to a specific evolutionary regimen with positive diversifying selection and concerted evolution that allows for rapid diversification of the binding region [39] and Fig. 9. The high level of polymorphism in the ligand-binding repeat region of NWD2 in wild-type isolates is compatible with a proposed role as immune receptor. The variability of *nwd2* in natural population might reflect a possible ligand-receptor arms race between *nwd2* and its ligand(s). Analysis of variability of the NWD2 homologs in different species (S2 Data) reveals that the C-terminal region is not always composed of highly conserved WD-repeats as found in *Podospora*, in certain cases Ankyrin repeats are found, in other species no typical repeat regions are found, raising the possibility that NWD2 ligands differs in different species.

### The Origin of the [Het-s] Prion

[Het-s] is highly prevalent in nature and [Het-s]/HET-S incompatibility was found to limit transfer of deleterious senescence plasmids between strains [14]. HET-s and HET-S are non-equivalent interaction partners that trigger a cell death reaction in which [Het-s] prion seeds activate the HET-S cell-death execution module acting as a the pore-forming toxin [17]. The evolutionary origin of this functional prion system has long been a puzzle that the discovery of the functional interaction between HET-S and NWD2 can help to solve. The HET-S/NWD2 gene pair is conserved in a wide range of fungal species while, apparently, the [Het-s]/HET-S incompatibility system is specific to *Podospora anserina* [21,24]. HET-s can be stably propagated as a prion because a specific amino acid difference at position 33 of the HeLo domain abolishes pore-forming activity in response to PFD transconformation [15,17,37]. These observations support the proposal that the [Het-s]/HET-S incompatibility system derives from the pre-existing *nwd2/het-S* gene complex by exaptation [14,17]. An exaptation is an evolutionary process in which a gene or an organ is co-opted to perform a new function that was not originally the target of selection [70]. In that model, a point mutation of the HeLo domain of HET-S has allowed formation of the [Het-s] prion and as a consequence of the [Het-s]/HET-S incompatibility system [14,17,24]. In other terms, in this exaptation process, a toxin (HET-S) has been turned into a toxin activation trigger ([Het-s]). In the absence of a functional cell death execution module, *nwd2* becomes useless, and indeed, it turns out that in wild *het-s* strains, *nwd2* is a pseudogene [14]. From an epistemological point of view, it is interesting to note that discovery of the NWD2/HET-S signal transduction process derives from studies of an exaptation of this system in the form of [Het-s]/HET-S incompatibility.

### Prion and Amyloid Signaling in Host Defense Pathways in Mammals and Fungi

The formation of higher-order signaling machines has been put forward as a novel paradigm for signal transduction [71]. Such open-ended signaling complexes occur in particular in the context of innate immunity in mammals. Among those complexes are the Myddosome [72], the MAVS CARD filaments [73], the RIP1/RIP3 necrosome [74] and the ASC-dependent inflammasomes [75]. All these immune signaling complexes build into large filamentous assemblies. In the case of the RIP1/RIP3 complex, higher-order assembly formation relies on short amyloid-forming motifs termed RHIM [74]. For two domains of the death domain fold family (DD) involved in such processes, the CARD and the PYD domain, their polymerization and templating properties allows them to function as prion-forming domains in yeast [38]. These signaling machines function in a process that has been designated prion-like and that in some instances, but not in others, involves amyloid structures. Based on sequence analysis, we had previously proposed that NWD2 controls an amyloid-based signal transduction process [24] and Cai et al. provided the first experimental support for this hypothesis by showing that the NWD2 HET-s-like motif can functionally replace the PYD domain in the prion-like NLRP3-signaling pathway [38]. Prion-like signal transduction mechanisms thus appear to be conserved from fungi to humans, in particular in the context of NOD-like receptor proteins. Activated NLRP3 NOD-like receptor can nucleate PYD filaments of ASC in a prion-like mechanism [75], [38]. There is a key structural difference between signaling mediated by DD folds and the HET-S/s-like motif. PYD filaments are assembled from α-helical folded domains whose fold exists before oligomerization of the Nod-like receptor [75]. In the case of NWD2/HET-S signaling, because amyloid folding is strictly cooperative, the transduction process would correspond to the de novo formation of the HET-S/s-like fold. In one case, the process corresponds to the cooperative assembly of previously folded α-helical building blocks; in the second case the transduction process corresponds to the cooperative emergence of a cross-β fold. It has been stressed that the higher-order assembly mode of signal transduction endows signal transduction cascades with specific properties such as signal amplification, threshold response, and noise reduction [71]. Such properties might be particularly critical in the context of signaling pathways that engage cell fate. Response must be mounted efficiently, but inappropriate activation of a cell death execution module such as the HeLo pore-forming domain is to be avoided. The strictly cooperative nature of amyloid folds and their prion-templating properties make such motifs particularly suited for ensuring such signal transduction tasks. Amyloid signaling based on motifs such as the HET-S/s-motif might also offer the advantage of extreme compactness compared to α-helical folded domains such as PYD or CARD. Several fungal species encode Nod-like receptors with N-terminal HeLo domains, suggesting that amyloid-mediated signaling is not strictly required for HeLo domain Nod-like receptor function in fungi [24,34]. It remains to be determined whether or not this all-in-one architecture predates the more complex two-component architecture involving an amyloid adaptor motif.

This study supports the unified role of higher-order complexes in signal transduction cascades and illustrates a specific case in which such a transduction processes involves a short amyloid motif that defines a well ordered structure with templating activity. Fungal genomes encode several other amyloid motifs located at the N-terminus of Nod-like proteins and at the C-terminus of their putative downstream effector proteins thus underlying the evolutionary success of this mode of signal transduction in fungi [20,24].

## Materials and Methods

### Prion Propagation, Prion Formation, and Incompatibility Assays

Methods for determination of incompatibility phenotypes, prion formation, and prion propagation were as previously described [76]. In brief, incompatibility phenotypes were determined by confronting strains on solid corn meal agar (DO) medium to [Het-s] and [Het-S] tester strains and visualizing the formation of barrages (abnormal contact lines forming upon confrontation of incompatible strains). [Het-s] prion propagation was assayed as the ability to transmit the [Het-s] prion phenotype from a donor strain to a [Het-s*] prion-free tester strain after confrontation on solid medium.

### Protein Transfection Assay

All synthetic peptides were synthesized by GENOSPHERE Biotechnologies (France) at purity >95%. Fibrils formation was carried at room temperature and occurred spontaneously after solubilising the synthetic peptides in Milli-Q water at concentration of 2.5 mg ml^-1^. Protein transfection experiments were performed as described previously using a cell disruptor (Fast-prep FP120, Bio101, Qbiogen Inc.) [48,52,76]. For each test, ~0.5 cm^3^ of [Het-s*] mycelium grown on solid medium is sheared (run time 20 s, 6 m.s^-1^) in 450 l of STC50 buffer (0.8 M sorbitol, 50 mM CaCl_2_, 100 mM Tris HCl pH 7.5) and amyloids of synthetic peptides or HET-s(218–289) assembled as described above (50 l at 2.5 mg ml^-1^) in a 2 ml screw-cap tube. The sheared mycelium is then plated onto DO medium (70 μl by spot at distance of 2 cm of [Het-S] strain) and incubated at 26°C until direct confrontation with the [Het-S] strain occurred (5 d) and the number of strains producing a barrage reaction to [Het-S] were counted.

### Transmission Electron Microscopy, FTIR Spectroscopy, Amyloid Seeding Assay

For negative staining, aggregated proteins were adsorbed to carbon-coated grids, rinsed with water, and stained with 2% (w/v) uranyl acetate. The samples were imaged in a Hitachi H-7000 transmission electron microscope (TEM) operating at an accelerating voltage of 75 kV.

Attenuated total reflectance (ATR) FTIR analysis was performed using a Bruker Tensor 27 FTIR Spectrometer (Bruker Optics, Inc.) with a Golden Gate MKII ATR accessory. Each spectrum consists of 20 independent scans, measured at a spectral resolution of 2 cm^-1^ within the range 1,800–1,500 cm^-1^. All spectral data were acquired and normalized using the OPUS MIR Tensor 27 software. Second derivatives of the spectra were used to determine the frequencies at which the different spectral components were located.

HET-s(218–289) C-terminally tagged with 6x-histidine, Sup35 NM fragment (residues 1–254) C-terminally tagged with 7x-histidine, the full-length Ure2p N-terminally tagged with 6x-histidine, and PrP (23–231) N-terminally tagged with 6x-histidine were expressed and purified essentially as described previously. Amyloid formation was performed at 100 M in the conditions described previously [52,77–81]. HET-s(218–289) aggregation assays were carried out as described previously [55,67] with of 15 M HET-s(218–289) at pH 7 and 37°C under high agitation (1,400 rpm using a Thermomixer, Eppendorf, Hamburg, Germany) in presence or absence of 1.5 M of pre-aggregated amyloid seeds.

### Solid-State NMR

Solid-state NMR experiments were performed on a 500 MHz ^1^H Larmor frequency spectrometer (Bruker Biospin, Germany) using a double resonance H-X probehead at a magic-angle spinning rate of 7 kHz. The sample temperature was set to 6°C with the internal reference DSS [82]. A ramped CP (cross-polarization) with 1 ms contact time was used for the ^1^H-^13^C polarization transfer. The subsequent ^13^C-^13^C polarization transfer was achieved via a proton-driven spin-diffusion (PDSD) with a mixing time of 25 ms to correlate intra-residual carbon atoms. Proton decoupling with a frequency of 85 kHz was applied during acquisition, using the SPINAL-64 decoupling sequence [83]. Spectra were analyzed and figures prepared using the CCPNMR analysis software [84].

## Supporting Information

S1 Data List of 55 HET-S homologs from various fungal species with the corresponding NWD2 homolog. (XLS)

S2 Data List of 24 NWD2 homologs used to generate the alignment and consensus sequence in Fig. 3. (XLS)

S3 Data Table including all numerical values that were used to generate graphs. (XLS)

S1 Fig Mutations predicted to affect ATP binding in the NACHT domain of NWD2 abolish [Het-s] inducing activity. [Het-s]-inducing activity of wild-type and mutated NWD2^e1^ alleles in a *ΔPaHsp104 het-c2* background. Experiments have been carried out at least in triplicate and error bars are standard deviations; the asterisks denote that prion conversion rate was zero.
(PDF)

S2 Fig Expression of NWD2(1–30)-GFP mutants. A. Strains transformed with wild-type and mutant NWD2(1–30)-GFP constructs were analyzed by western-blotting with anti-GFP and control anti-tubulin antibodies as marked. A strain expressing GFP was also used as control. For each strain, the signal corresponding to the upper band revealed by the anti-GFP antibody was quantified, the lower band at the size of the GFP likely corresponds to a degradation product in which the NWD2(1–30) region was cleaved. For each mutant, the ratio of the amount of detected NWD2(1–30)-GFP with respect to wild-type is given in percentage. The amount of mutant protein is at least that of wild-type, except for H9P and G19A. B. The same experiment was performed this time with two different wild-type transformants and three different H9P and G19A transformants. Note that expression level differs in each transformant as transformation leads to multicopy integration at ectopic sites. As above, the ratio of the amount of detected NWD2(1–30)-GFP with respect to wild-type (strain 1) is given in percentage. Amounts of H9P and G19A are close to wild-type strain 1. While expression levels vary between transformants, all mutant constructs are expressed, making it unlikely that lack of [Het-s]-inducing activity is due to lack of expression. Only G19A shows an expression level slightly below wild-type in the analyzed transformants.
(PDF)

S3 Fig NWD2(1–30)-GFP leads to an incompatibility reaction with HET-S. Strains expressing NWD2(1–30)-GFP and H9P and G19A mutants were confronted to HET-S tester strains on solid medium. Barrage reaction occurs with wild type but not mutant NWD2(1–30)-GFP.
(PDF)

S4 Fig NWD2(1–30)-RFP causes HET-S-GFP localization to the cell periphery. Full panels for the zoomed images presented in Fig. 6 are given. For each image, the zoomed region is boxed in white on the DIC and GFP/RFP merged image.
(PDF)

S1 Methods Supplementary methods. (DOC)

S1 Table Genotype of *het-S* wild-isolates for the *nwd2* and *het-c* genes. (PDF)

S2 Table *nwd2–8* shows no inducing activity in *het-c1*, *het-c2*, and *het-c4* backgrounds. (PDF)

## Abbreviations

CP: Cross-polarization
DD: death domain fold family
FTIR: Fourier Transform InfraRed spectroscopy
GFP: Green Fluorescent Protein
INEPT: Insensitive Nuclei Enhanced by Polarization Transfer
MAS: magic angle spinning
NLR: Nod-like receptor
NOD: nucleotide-binding and oligomerization domain
PFD: prion forming domain
RFP: Red Fluorescent Protein
STAND: Signal Transduction ATPase with Numerous Domains
