# Supplementary figures and images for: Signal Transduction by a Fungal NOD-Like Receptor Based on Propagation of a Prion Amyloid Fold
Source: PLoS Biol. 2015 Feb 11;13(2):e1002059. doi: 10.1371/journal.pbio.1002059 (PMC4344463; doi:10.1371/journal.pbio.1002059)

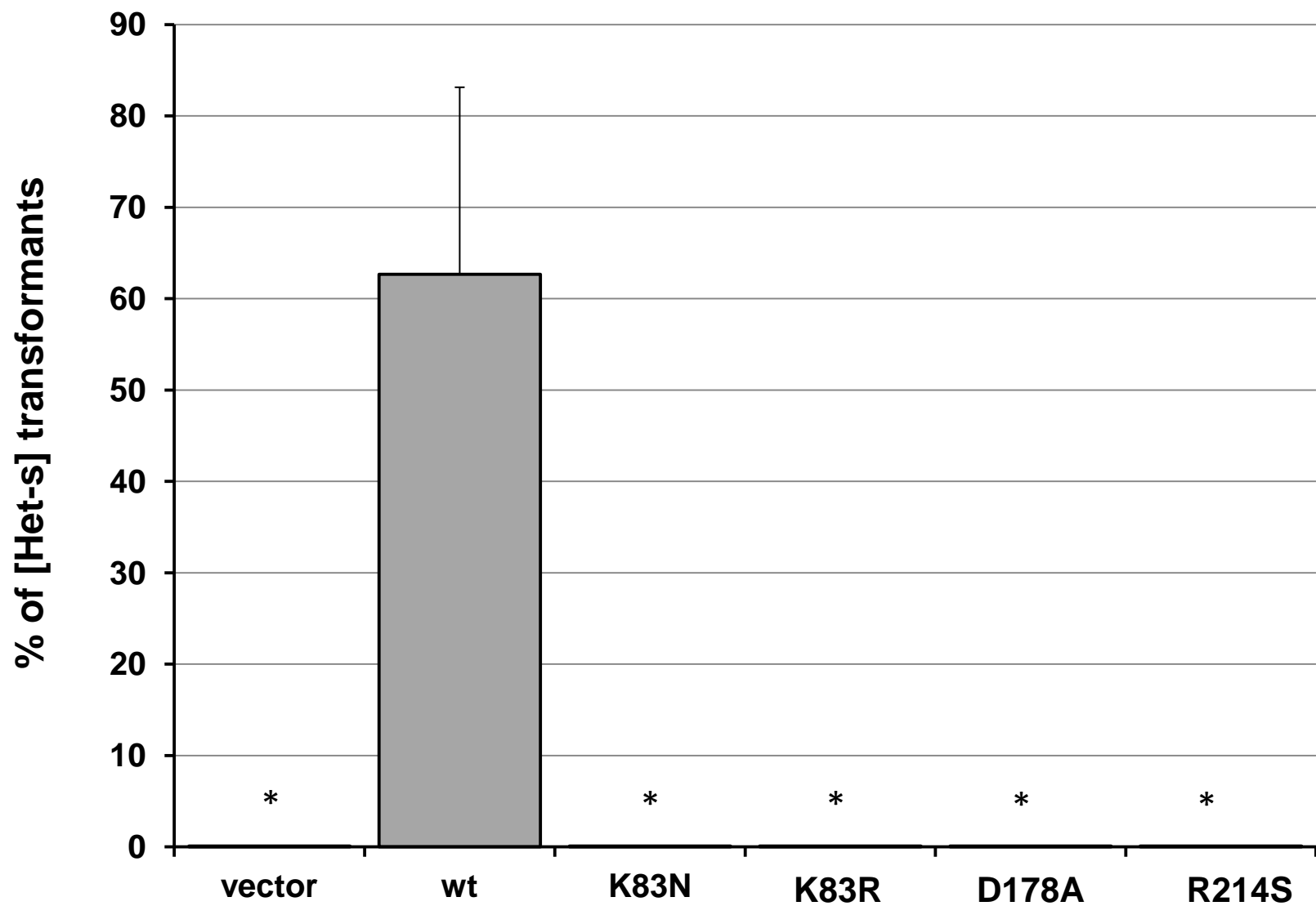

Supplement: S1 Fig — [Het-s]-inducing activity of wild-type and mutated NWD2e1 alleles in a ΔPaHsp104 het-c2 background. Experiments have been carried out at least in triplicate and error bars are standard deviations; the asterisks denote that prion conversion rate was zero. (PDF) [file pbio.1002059.s004.pdf]

# A

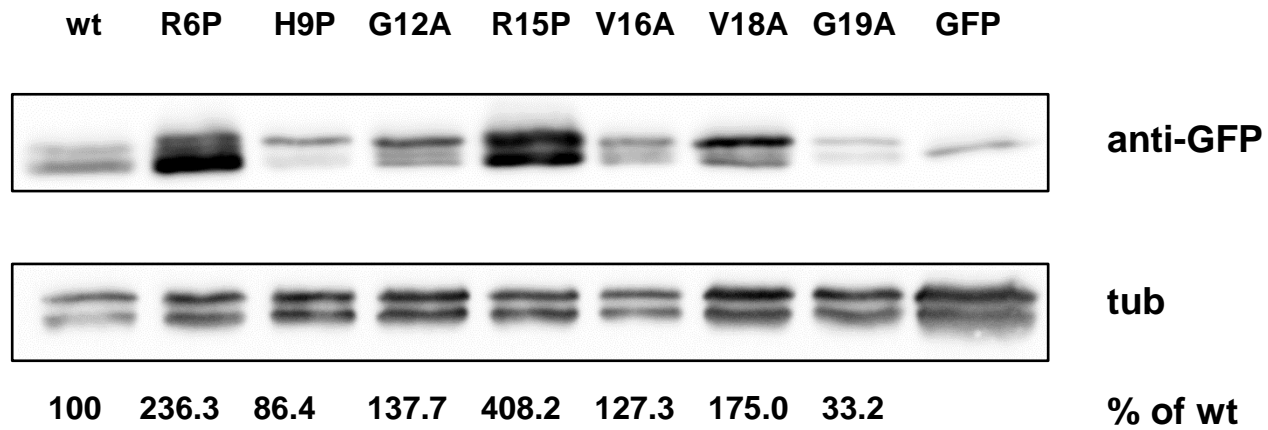

# B

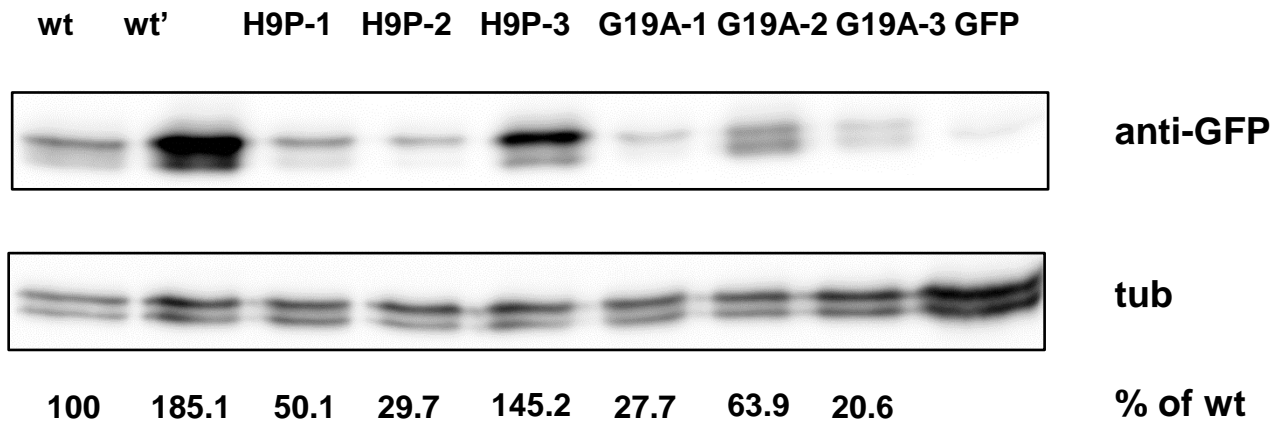

Supplement: S2 Fig — A. Strains transformed with wild-type and mutant NWD2(1–30)-GFP constructs were analyzed by western-blotting with anti-GFP and control anti-tubulin antibodies as marked. A strain expressing GFP was also used as control. For each strain, the signal corresponding to the upper band revealed by the anti-GFP antibody was quantified, the lower band at the size of the GFP likely corresponds to a degradation product in which the NWD2(1–30) region was cleaved. For each mutant, the ratio of the amount of detected NWD2(1–30)-GFP with respect to wild-type is given in percentage. The amount of mutant protein is at least that of wild-type, except for H9P and G19A. B. The same experiment was performed this time with two different wild-type transformants and three different H9P and G19A transformants. Note that expression level differs in each transformant as transformation leads to multicopy integration at ectopic sites. As above, the ratio of the amount of detected NWD2(1–30)-GFP with respect to wild-type (strain 1) is given in percentage. Amounts of H9P and G19A are close to wild-type strain 1. While expression levels vary between transformants, all mutant constructs are expressed, making it unlikely that lack of [Het-s]-inducing activity is due to lack of expression. Only G19A shows an expression level slightly below wild-type in the analyzed transformants. (PDF) [file pbio.1002059.s005.pdf]

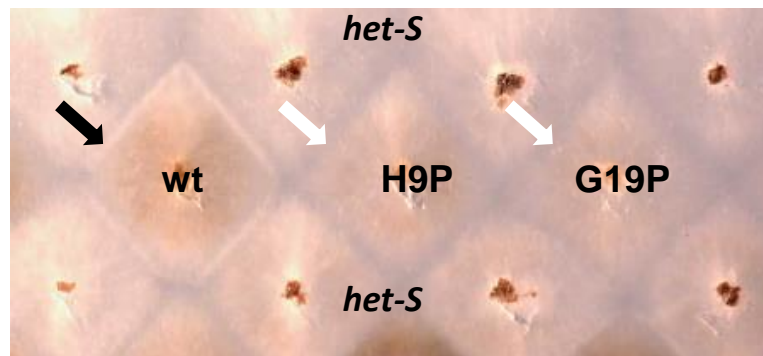

Supplement: S3 Fig — Strains expressing NWD2(1–30)-GFP and H9P and G19A mutants were confronted to HET-S tester strains on solid medium. Barrage reaction occurs with wild type but not mutant NWD2(1–30)-GFP. (PDF) [file pbio.1002059.s006.pdf]

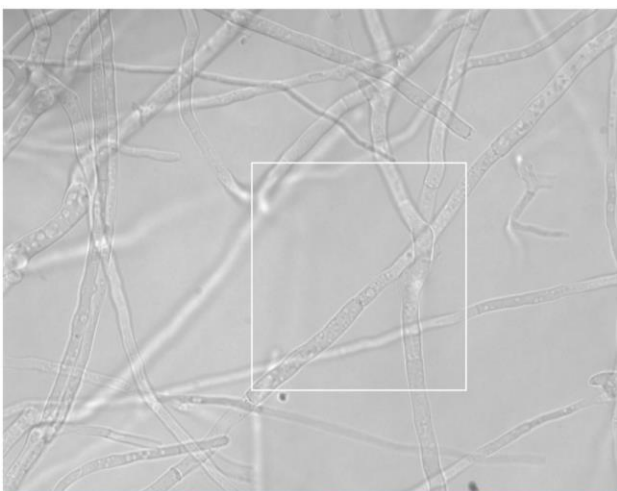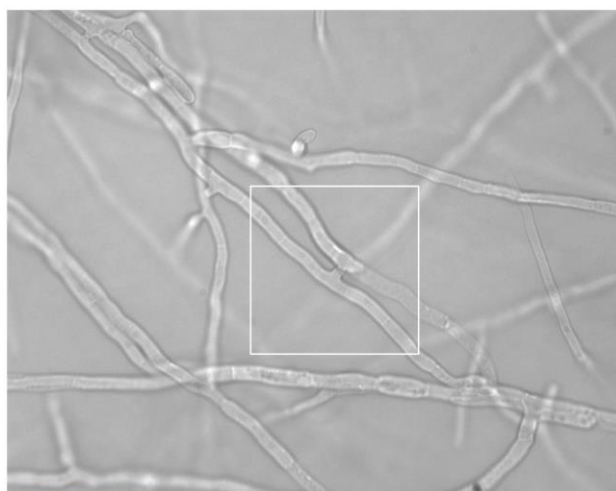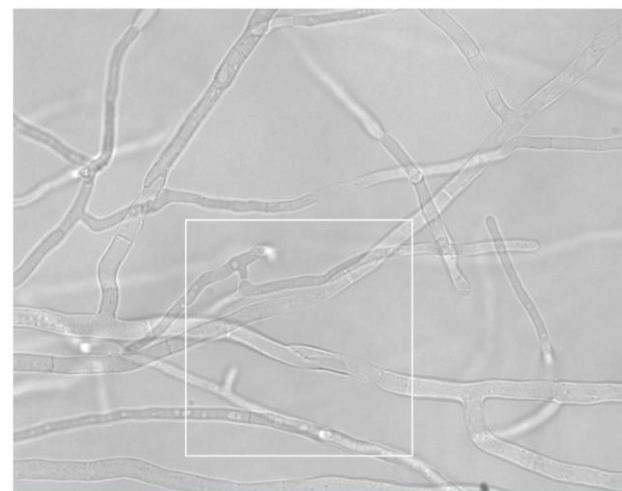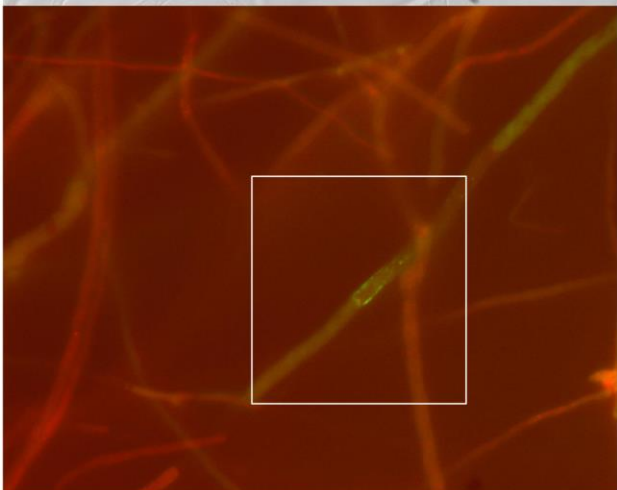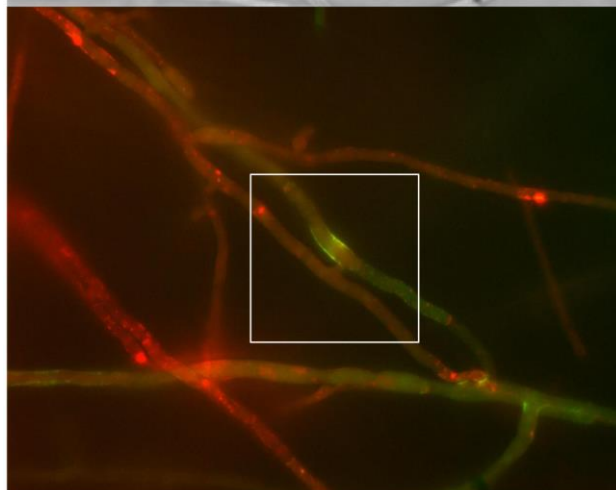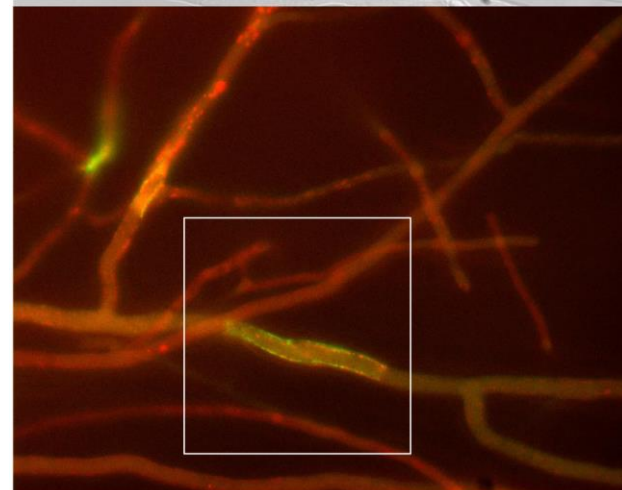

Supplement: S4 Fig — Full panels for the zoomed images presented in Fig. 6 are given. For each image, the zoomed region is boxed in white on the DIC and GFP/RFP merged image. (PDF) [file pbio.1002059.s007.pdf]
