# Supplementary material for: Signal Transduction by a Fungal NOD-Like Receptor Based on Propagation of a Prion Amyloid Fold
Source: PLoS Biol. 2015 Feb 11;13(2):e1002059. doi: 10.1371/journal.pbio.1002059 (PMC4344463; doi:10.1371/journal.pbio.1002059)
Supplement: S1 Methods — (DOC) [file pbio.1002059.s008.doc]

**Supplementary methods**

**Strains, plasmids and media**

*P. anserina* strains used in this study were wild-type *het-s*, *het-S,* *Δhet-s* strains and the *ΔPaHsp104* strain *het-c1, het-c2, het-c3 and het-c4* strains and a wild-type *het-c2* strain and *het-e1*, *het-d1* and *het-d2* strains . Growth medium for barrage assays and prion transmission assays was standard corn meal agar DO medium. The three chimeric constructs *nwd2^e1^*, *nwd2^d1^* and *nwd2^d2^* were obtained by amplifying the region encoding the amino acid *1-543* of the *nwd2* gene including its promoter using oligonucleotides 5’ CGGGATCCCGCATGGCAGTTTGACGGGAAAGCGAAGGTTGATG 3’ and 5’ GGGGTACCCTAAAGGCGCGTAACATACATGTAG 3’ and using genomic DNA of *het-e1*, *het-d1* and *het-d2* strains as template. The PCR products were cloned as *Bam*HI *and Kpn*I fragments in the pUC18 vector to yield pUC18-nwd2(1-543). The region encoding the C-terminal WD-domain of *het-e1, het-d1 and het-d2* including their terminator were amplified by PCR using oligonucleotides 5’ GGGGTACCTGAGACCACTAAATGCTATAGGC 3’ and 5’ GGAATTCTCTAGACTCATCGACTCCTTATGCGTTTG 3’ for *het-e1*, and 5’ ATGCGGTACCCTACCCAGTAATAGTGTCCTAT 3’ and 5’ ATGCGAATTCACGGCAAACAAGCAATCAGC 3’ for *het-d1* and *het-d2.* The PCR products were cloned in the pUC18-nwd2(1-543) vector downstream of *nwd2(1-543)* using *Kpn*I*and Eco*RI restriction sites to yield the plasmids pUC-nwd2^e1^ , pUC-nwd2^d1^ and UC-nwd2^d2^ used in the prion induction assays.

The pOP-nwd2(1-30)-GFP plasmid allowing expression of the NWD2(1-30) in *P. anserina* was generated by inverse PCR using as template the pOP-nwd2^e1^-GFP plasmid with oligonucleotides 5’ ATATGGTACCATGGTGAGCAAGGGCGAGG 3’ and 5’ ATATGGTACCCGGGAGGAAGTCGCTCGAGCC 3’ and the PCR product was digested and re-ligated using a *Kpn*I restriction site. The expression of *nwd2(1-30)-GFP* is under the control of a constitutive *gpd* (glyceraldehyde-3-phosphate dehydrogenase) Yeast experiments were carried out with strain L1749 containing the pHet-s(PrD)-GFP-*TRP1* plasmid allowing expression of HET-s(218-289)-GFP from a *gal* promotor . Yeast was grown at 30°C on synthetic glucose media lacking tryptophan and uracil (SD-Trp,Ura) or synthetic media lacking Trp and Ura containing 2% raffinose and 0.05% galactose (SG 0.05% -Trp,Ura) to induce a low expression of HET-s(218-289)-GFP yeast cells were grown in SD–Trp, Ura containing 0.05% galactose and 2% raffinose (SG 0.05% -Trp,Ura). For the expression of the N-terminus of NWD2(1-543) in yeast we have used the pTG-NWD2(1-543)-*URA3* *cen* plasmid. The expression of NWD2 is under the constitutive promoter of the Phosphoglycerate kinase encoding gene (PGK). The DNA fragment of *nwd2(1-543)* was amplified by PCR using oligonucleotides 5’ ATGCAGATCTATGGCAAATCAGGTTCGGTC 3’ and 5’ ACGGTCGACCTAATGTAGAAAGGTTAATAAGTC 3’ and cloned into pTG887 using *Bgl*II and *Sal*I restriction sites.

**Ligand-induced prion templating assay**

Plasmids (pUC-nwd2^e1^ pUC-nwd2^d1^ and pUC-nwd2^d2^) expressing the chimeric *nwd2* alleles were used with the pPa-Ble plasmid to co-transform *P. anserina* [Het-s*] *Δhsp104 het-c1, 2, 3 and 4* strains. Plasmid ratios were between 1:3 and 1:5 in favour of the plasmids expressing the NWD2 chimeras. After 3-5 days of regeneration at 26°C individual transformants were selected and tested for the [Het-s] prion phenotype. Transformants were confronted to a *het-S* strain on a standard corn meal agar DO medium and 24h after confrontation transformants showing [Het-s] prion phenotype (producing a barrage reaction) were counted. Experiments were carried out in triplicates or more except in the case of the *het-c1* and *het-c3* backgrounds that were tested in duplicates only. For each background and for each chimeric allele between 60 and 369 transformants were tested.

**Microscopy**

For fluorescence microscopy, synthetic medium containing 2 % (wt/vol) agarose was poured as two 10 ml layers of medium. *P. anserina* hyphae were inoculated on this medium and cultivated for 24 to 48 h at 26°C. The top layer of the medium was then cut out and the mycelium was examined with a Leica DMRXA microscope equipped with a Micromax CCD (Princeton Instruments) controlled by the Metamorph 5.06 software (Roper Scientific). The microscope was fitted with a Leica PL APO 100X immersion lens. Yeast cells were analysed with the same equipment. Cells from liquid cultures were spotted on solid (SG 0.05% -Trp,Ura) medium for microscopy observation.

**Protein extracts and western-blotting**

Total-protein extracts were prepared from strains grown on solid (SU) medium for 48h at 26 C°. Protein extraction was realized in denaturing conditions (Urea 9M, SDS 1%, Tris HCl pH 6.8 25 mM, EDTA 1mM, β-mercaptoethanol 0.7 M) after freeze-drying of the samples. Electrophoresis (SDS-PAGE) was performed using standard procedures on 12% polyacrylamid gels. Proteins were transferred (2h at 40 mA) to Amersham Hybond-ECL membrane (GE Healthcare) and immunoblotting was carried out with anti-GFP (Roche) and anti-TAT1 (Sigma) antibodies in PBS-T buffer (PBS 1x, Tween-20 0.1% w/v). Chemifluorescence (Amersham ECL Prime Western Blotting Detection Reagent) was recorded with Syngene PXi multi-application image analysis system and data quantification performed with ImageJ processing tool.
