# Supplementary material for: Signal Transduction by a Fungal NOD-Like Receptor Based on Propagation of a Prion Amyloid Fold
Source: PLoS Biol. 2015 Feb 11;13(2):e1002059. doi: 10.1371/journal.pbio.1002059 (PMC4344463; doi:10.1371/journal.pbio.1002059)
Supplement: S1 Table — (PDF) [file pbio.1002059.s009.pdf]

Table S1. Genotype of *het-S* wild-isolates for the *nwd2* and *het-c* genes

strain	*nwd2* allele	*het-c* allele	*het-c* phenotypic class
Wa7	*nwd2-8*	*het-c2*	C2
Wa11	*nwd2-7*	*het-c2*	C2
Wa13	*nwd2-2*	*het-c2*	C2
Wa15	*nwd2-7*	*het-c5*	C1
Wa20	*nwd2-7*	*het-c2*	C2
Wa21	*nwd2-3*	*het-c7*	C7
Wa25	*nwd2-4*	*het-c1*	C1
Wa28	*nwd2-8*	*het-c2*	C2
Wa43	*nwd2-9*	*het-c5*	C1
Wa44	*nwd2-8*	*het-c2*	C2
Wa46	*nwd2-8*	*het-c3*	C3
Wa47	*nwd2-8*	*het-c3*	C3
Wa62	*nwd2-7*	*het-c2*	C2
Wa63	*nwd2-3*	*het-c9*	C9
Wa67	*nwd2-6*	*het-c5*	C1
Wa69	*nwd2-7*	*het-c1*	C1
Wa71	*nwd2-8*	*het-c2*	C2
Wa72	*nwd2-8*	*het-c2*	C2
Wa76	*nwd2-8*	*het-c2*	C2
Wa78	*nwd2-4*	*het-c1*	C1
Wa79	*nwd2-4*	*het-c1*	C1
Wa85	*nwd2-10*	*het-c2*	C2
Wa86	*nwd2-8*	*het-c6*	C3
Wa88	*nwd2-7*	*het-c2*	C2
Wa93	*nwd2-7*	*het-c2*	C2
Wa94	*nwd2-7*	*het-c2*	C2
Wa95	*nwd2-8*	*het-c3*	C3
Wa99	*nwd2-8*	*het-c5*	C1
Wa101	*nwd2-5*	*het-c2*	C2
Wa102	*nwd2-3*	*het-c1*	C1
Wa105	*nwd2-5*	*het-c1*	C1
Wa114	*nwd2-4*	*het-c2*	C2
