# Supplementary material for: Signal Transduction by a Fungal NOD-Like Receptor Based on Propagation of a Prion Amyloid Fold
Source: PLoS Biol. 2015 Feb 11;13(2):e1002059. doi: 10.1371/journal.pbio.1002059 (PMC4344463; doi:10.1371/journal.pbio.1002059)
Supplement: S2 Table — (PDF) [file pbio.1002059.s010.pdf]

Table S2. *nwd2-8* shows no inducing activity in *het-c1*, *het-c2* and *het-c4* backgrounds

	het-c1	*het-c2*	*het-c4*
negative control	0 [Het-s]/36 tested	0/60	0/90
$nwd2^{e1}$	nd.	25/59	0/90
$nwd2^{d2}$	nd.	0/60	100/140
nwd2-8	0/59	2/90	1/30
